# A degradable multi-metal-chelating stealth nanoplatform for dual ferroptosis/cuproptosis-enhanced metalloimmunotherapy in leukemia

**DOI:** 10.1186/s12951-026-04295-4

**Published:** 2026-03-21

**Authors:** Yingying Wang, Jianxiang Xu, Wenhui Bai, Ziwei Zhang, Chunmin Ma, Yayue Tan, Zhenge Zhang, Wanting Liu, Yunzhao Wu, Junchao Liu, Hu Lei, Hanzhang Xu, Wei Weng, Mei Huang, Xiaoyang Feng, Limin Zhu, Li Yang, Qi Zhu, Ying-Li Wu

**Affiliations:** 1 Faculty of Basic Medicine, Chemical Biology Division of Shanghai Universities E-Institutes, Key Laboratory of Cell Differentiation and Apoptosis of the Chinese Ministry of Education, Hongqiao International Institute of Medicine, Shanghai Tongren Hospital, Shanghai Jiao Tong University School of Medicine, Shanghai, 200025 China; 2 College of Biological Science and Medical Engineering, Shanghai Engineering Research Centerof Nano-Biomaterials and Regenerative Medicine, Donghua University, Shanghai, 201620 People’s Republic of China; 3 Yusuf Hamied Department of Chemistry, University of Cambridge, CB2 1EW Cambridge, UK; 4 Department of Respiratory and Critical Care Medicine, Shanghai Chest Hospital, School of Medicine, Shanghai Jiao Tong University, Shanghai, China; 5 Department of Oral and Maxillofacial-Head and Neck Oncology, Ninth People’s Hospital, College of Stomatology, School of Medicine, Shanghai Jiao Tong University, Shanghai, 200011 China; 6 Department of Vascular Surgery, Shanghai Ninth People’s Hospital, Shanghai JiaoTong University, School of Medicine, Shanghai, China; 7 Department of Rehabilitation, Shanghai General Hosptial, Shanghai Jiaotong University, No. 100, Haining Road, Shanghai, 200080 China

## Abstract

Acute myeloid leukemia (AML) remains a challenging hematologic malignancy with limited treatment options and poor prognosis. Here, we report the development of a multifunctional, pH-responsive, and biodegradable nanoparticle system, Membrane/Cu-HMPB@DSF/RSL3, for synergistic AML therapy. Constructed upon the Prussian blue-based frameworks and cloaked with leukemia cell membranes, these nanoparticles preferentially accumulate in AML cells and release copper, iron, and manganese ions, along with disulfiram (DSF) and RSL3, under mildly acidic intracellular conditions. The released metal ions catalyze Fenton-like reactions, deplete intracellular glutathione (GSH), and induce ferroptosis and cuproptosis in cooperation with the loaded small-molecule drugs. Meanwhile, manganese ions activate the cGAS-STING pathway, triggering innate immune responses and promoting immune cell recruitment. Both in vitro and in vivo studies demonstrated robust anti-AML efficacy with minimal systemic toxicity. This work presents a modular and immunogenic nanoplatform that holds broad potential for AML treatment and beyond.

**Graphical abstract:**

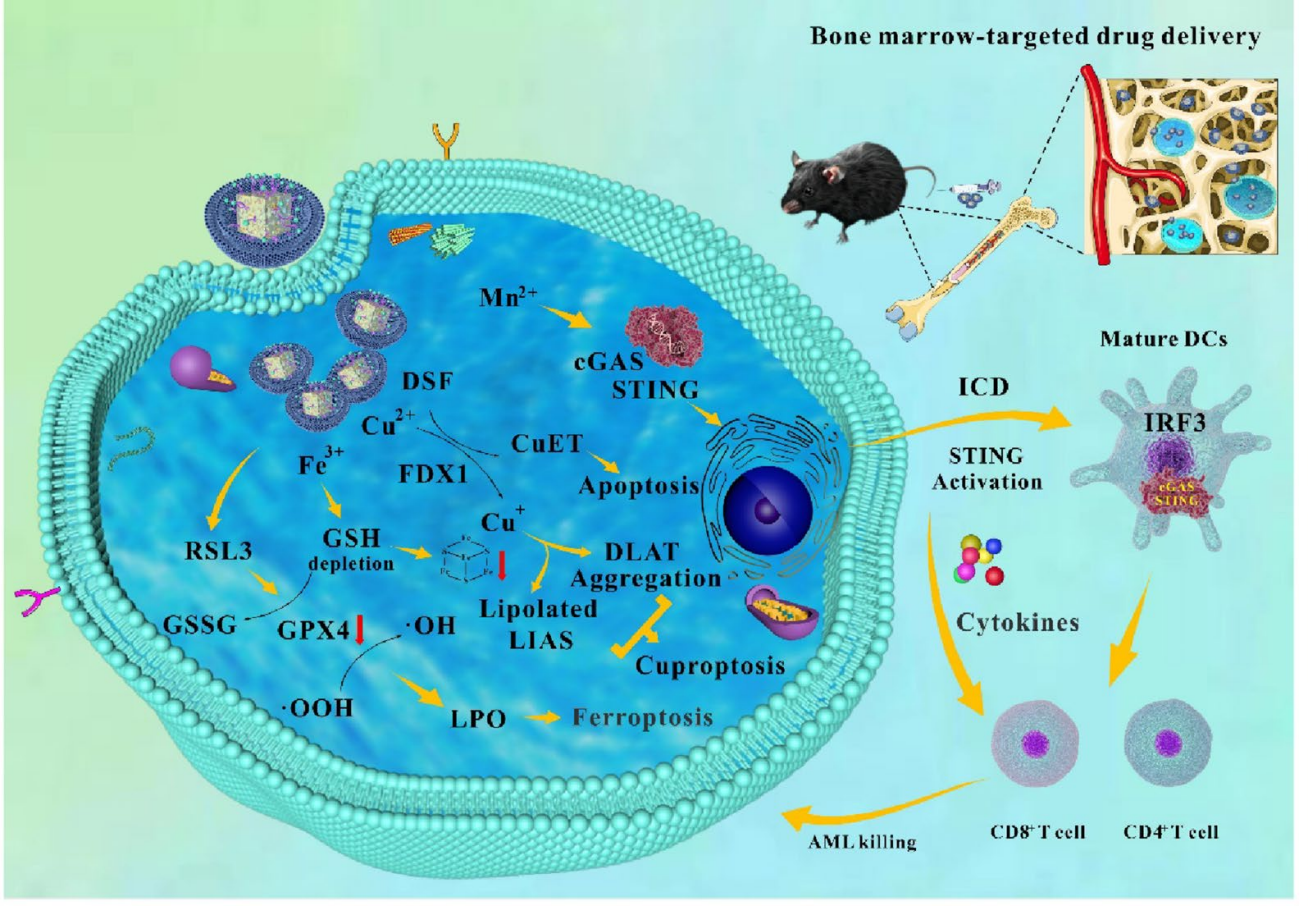

**Supplementary Information:**

The online version contains supplementary material available at 10.1186/s12951-026-04295-4.

## Introduction

Acute myeloid leukemia (AML) is a heterogeneous hematological malignancy characterized by the rapid proliferation of immature myeloid cells in the bone marrow and peripheral blood [1, 2]. Although conventional chemotherapy remains the standard of care, it faces substantial limitations, as over 50% of patients eventually develop chemoresistance and relapse [3–5]. Recent therapeutic innovations, including targeted therapies such as FLT3 inhibitors and venetoclax [6–9], have shown promise but remain limited due to the rapid development of drug resistance and the intrinsic heterogeneity of AML [10]. Allogeneic hematopoietic stem cell transplantation (HSCT) represents the only potentially curative approach; however, its application is highly limited: in 2016, only 12.6% of AML patients worldwide received HSCT due to scarcity of suitable donors, with rates dropping below 5% in underdeveloped regions [11]. Even among recipients, nearly half experience disease relapse [12]. These clinical challenges highlight the urgent need for more effective therapeutic strategies, particularly those capable of simultaneously targeting multiple pathways to eradicate leukemia cells and overcome resistance.

Recent discoveries in regulated cell death pathways have unveiled novel opportunities for AML therapy [13, 14]. Ferroptosis, an iron-dependent form of programmed cell death characterized by glutathione (GSH) depletion and lipid peroxidation (LPO), and cuproptosis, a copper-triggered cell death involving FDX-mediated mitochondrial proteotoxic stress, have both shown therapeutic potential [13, 16]. AML cells are particularly vulnerable to ferroptosis due to their elevated intracellular iron concentrations and oxidative stress levels [17, 19]. Similarly, disrupted copper homeostasis in leukemic cells renders them vulnerable to cuproptosis [20, 21]. Interestingly, these two pathways share mechanistic overlaps: both iron and copper ions can generate reactive oxygen species (ROS) through Fenton-like reactions [22], and both ultimately disrupt mitochondrial function. Ferroptosis alters mitochondrial morphology, and cuproptosis interferes with mitochondrial protein lipoylation, which further amplifies oxidative stress and enhances ROS production 

[23, 24]. These mechanisms suggest that concurrent activation of ferroptosis and cuproptosis may exert synergistic cytotoxic effects. Mounting evidence has revealed extensive crosstalk between ferroptosis, cuproptosis, and other cell death pathways [25, 26], suggesting that strategically manipulating these interconnected pathways could substantially enhance the therapeutic outcomes in AML.

In parallel with direct cytotoxic treatments, immunotherapy transformed the landscape of cancer treatment by leveraging the host immune system. The cGAS-STING pathway, activated by aberrant accumulation of cytosolic double-stranded DNA (dsDNA), promotes type I interferon production and proinflammatory signaling, ultimately recruiting and activating immune effector cells to eliminate cancer [27, 28]. Divalent manganese ions (Mn^2+^) play a pivotal role in cGAS-STING activation by increasing cGAS sensitivity for dsDNA recognition and strengthening the binding between STING and 2’,3’-cyclic GMP-AMP (cGAMP), a critical second messenger in the cGAS-STING pathway [29, 30]. Moreover, Mn^2+^ has been shown to stimulate CD8^+^ T cell and NK cell activation, as well as dendritic cell (DC) maturation and antigen presentation [29]. Combining Mn^2+^ with standard chemotherapy or anti-PD-1 antibody treatment has shown significant clinical benefits for patients with advanced metastatic solid tumors [29]. Hence, incorporating Mn^2+^ could potentially enhance the treatment efficacy by activating anti-tumor immune responses alongside cell death pathways.

**Scheme 1:**
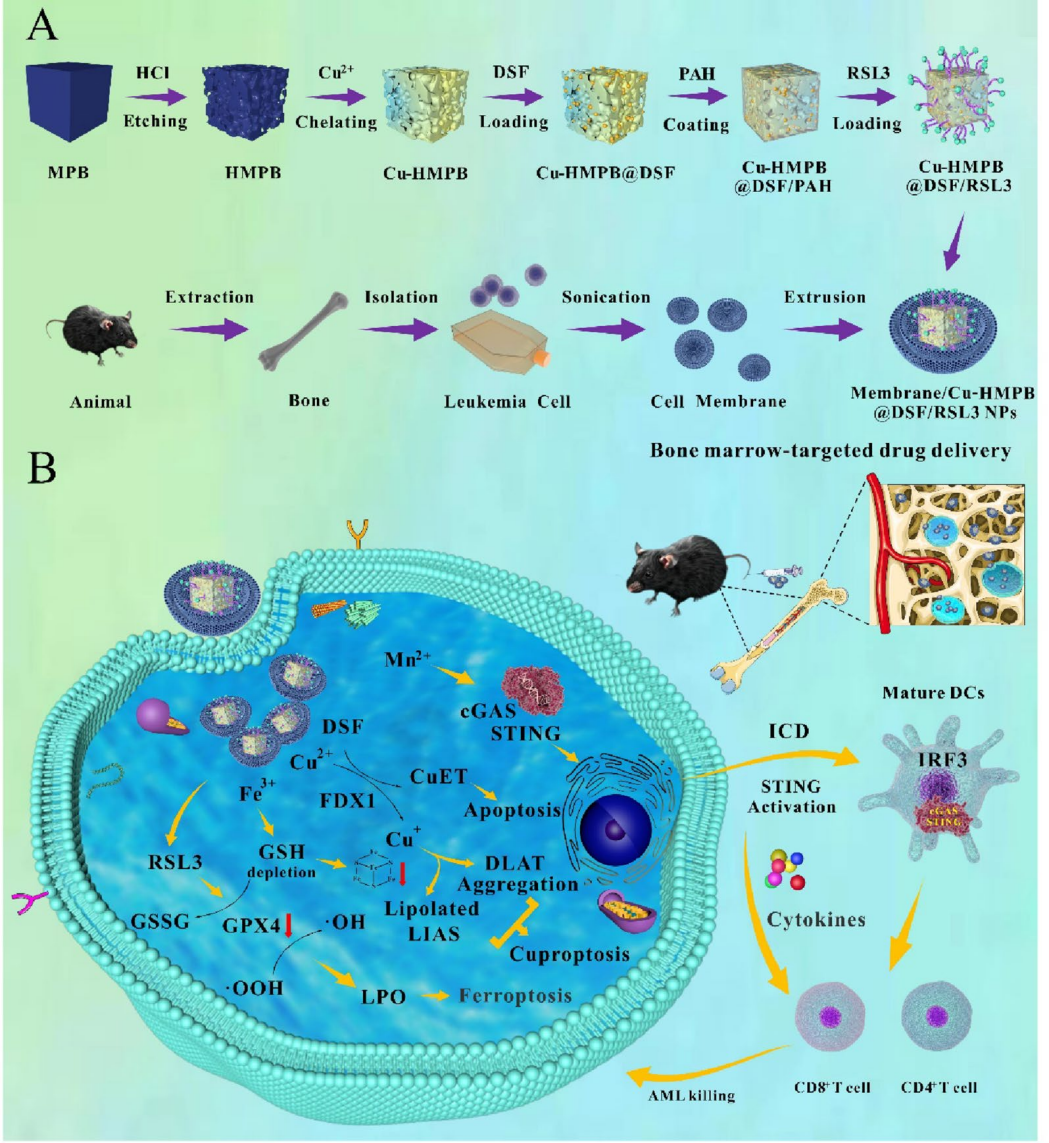
(**A**) A schematic of the synthesis process of NPs. (**B**) Proposed mechanisms of AML metalloimmunotherapy using Membrane/Cu-HMPB@DSF/RSL3 NPs. The anti-AML activity of the NPs is validated in vitro and in vivo

In this study, we designed a novel degradable multi-metal ion chelating nanoparticles (Membrane/Cu-HMPB@DSF/RSL3 NPs) that integrates metal ion-mediated cuproptosis and ferroptosis with immunotherapy (Scheme 1A). The core structure of the NPs consists of hollow mesoporous Prussian blue containing iron, copper, and manganese ions. The mesoporous Prussian blue framework is loaded with a small-molecule drug, disulfiram (DSF), then modified on the surface with the ferroptosis inducer RSL3 and coated with C1498 AML cell membranes. The AML cell membrane coating imparts homing capability, allowing the nanoparticles to evade macrophage-mediated clearance and specifically target AML cells. DSF acts as a copper ionophore to enhance Cu^2+^-induced cuproptosis, while RSL3 inactivates glutathione peroxidase 4 (GPX4), promoting LPO and inducing ferroptosis in AML cells. This process can deplete intracellular GSH, further synergizing with cuproptosis to enhance cytotoxicity. Following the immunogenic death of AML cells, released dsDNA collaborates with Mn^2+^ to activate the cGAS-STING pathway, enhancing anti-leukemic immune responses. After synthesis and characterization, we validated the anti-AML activity of these NPs through a series of in vitro and in vivo experiments (Scheme. 1B).

## Results

### Nanoparticle synthesis and characterization

**Fig. 1:**
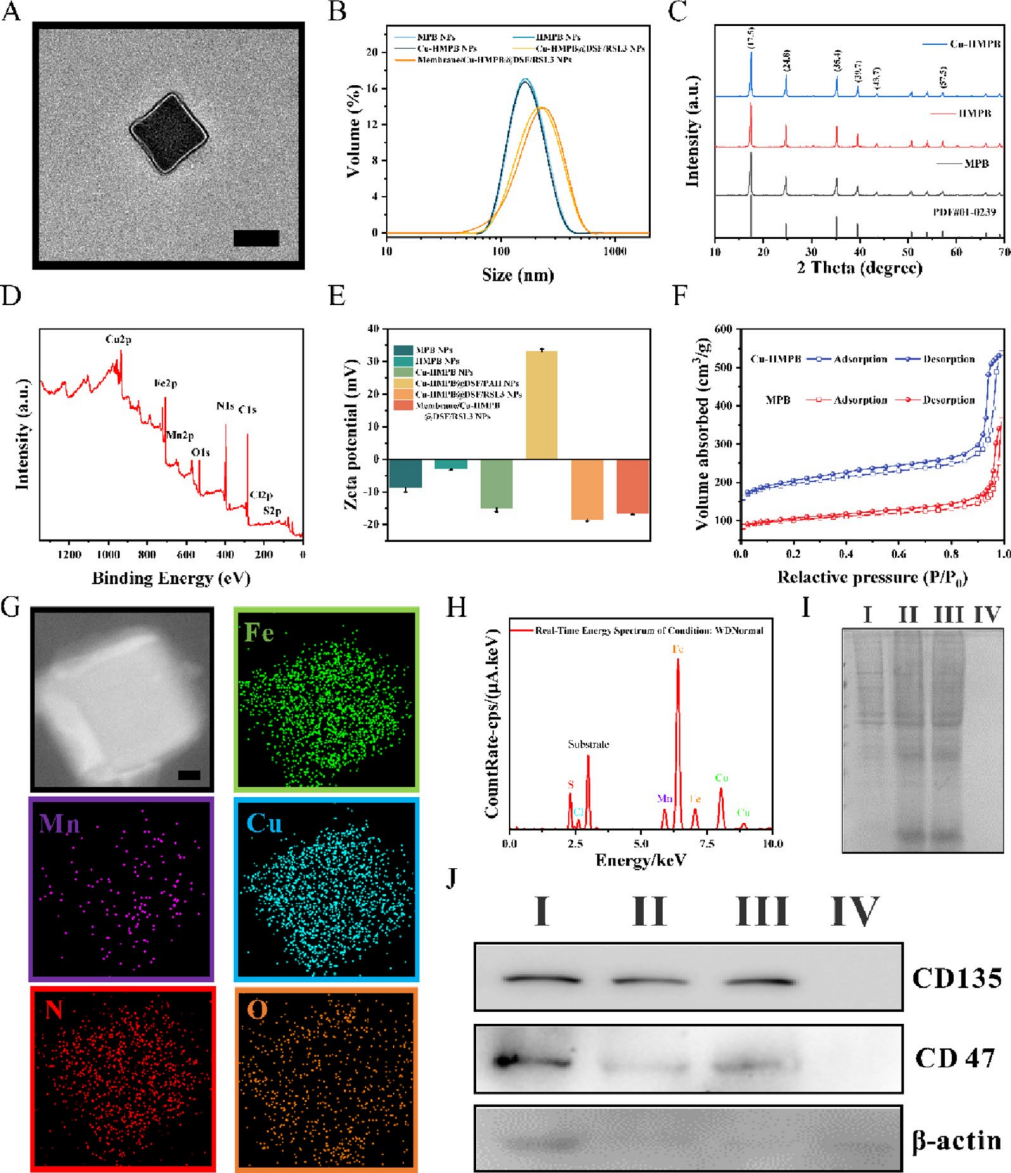
Characterization of membrane/Cu-HMPB@DSF/RSL3 NPs. (**A**) TEM image of a representative membrane/Cu-HMPB@DSF/RSL3 NPs. Scale bar: 100 nm. (**B**) Particle size distributions of MPB NPs, HMPB NPs, Cu-HMPB NPs, Cu-HMPB@DSF/RSL3 NPs, and membrane/Cu-HMPB@DSF/RSL3 NPs. (**C**) XRD patterns of MPB NPs, HMPB NPs and Cu-HMPB NPs. (**D**) High-resolution XPS spectra of Cu-HMPB@DSF/RSL3 NPs. (**E**) Zeta potential values of the indicated NPs. (**F**) N_2_ adsorption-desorption isotherm analysis of MPB NPs and Cu-HMPB NPs. (**G**) Element mapping of Cu-HMPB NPs by high-resolution transmission electron microscopy (HRTEM). Green, blue, violet, red, and orange correspond to Fe, Cu, Mn, N, and O element, respectively. Scale bar: 25 nm. (**H**) Metal element detection under the WDNormal condition. (**I**) SDS-PAGE analysis of protein profiles from AML cells and membrane/Cu-HMPB@DSF/RSL3 NPs. (**J**) Western blot confirms the presence of CD135 and CD47 proteins on the membrane/Cu-HMPB@DSF/RSL3 NPs surface (Ⅰ: cell lysate, Ⅱ: leukemia cell membrane, Ⅲ: membrane/Cu-HMPB@DSF/RSL3 NPs, Ⅳ: Cu-HMPB@DSF/RSL3 NPs)

Mn-containing Prussian blue nanoparticles (MPB NPs) were synthesized *via in situ* replacement of Fe^3+^ with Mn^2+^ during the reaction of Na_4_[Fe(CN)_6_]·10H_2_O with FeCl_3_·6H_2_O and MnCl_2_·4H_2_O in water. Transmission electron microscopy (TEM) analysis revealed an average particle size of ~150 nm (Fig. S1A). Hollow mesoporous HMPB NPs were subsequently prepared through controlled chemical etching, exhibiting a size of ~145 nm (Fig. S1B). Cu-HMPB NPs were formed by ion exchange between HMPB NPs and copper ions in the presence of polyvinylpyrrolidone (PVP), resulting in a size of ~147 nm (Fig. S1C). Retaining PVP on the NPs surface was essential for dispersion stability, as confirmed by FT-IR spectroscopy (Fig. S2). Subsequently, hydrophobic DSF was loaded into Cu-HMPB NPs, leading to an increased particle size of ~150 nm (Fig. S3A). Poly (allylamine hydrochloride) (PAH) coating enlarged the NPs to ~155 nm (Fig. S3B). Functionalization with RSL3, a ferroptosis inducer, further increased the size to ~165 nm (Fig. S3C). Finally, the Cu-HMPB@DSF/RSL3 NPs were encapsulated with C1498 AML cell membranes *via* extrusion, forming a ~ 7.5 nm membrane layer, reaching a final diameter of ~170 nm (Fig. 1A; Fig. S4A, S4B). As shown in Fig. 1B, the hydrodynamic particle sizes of MPB NPs, HMPB NPs, and Cu-HMPB NPs were 161 ± 11 nm. After cell membrane encapsulation, the hydrodynamic size of Membrane/Cu-HMPB@DSF/RSL3 NPs increased from 199 ± 24 nm to 219 ± 15 nm. Notably, the size of the Membrane/Cu-HMPB@DSF/RSL3 NPs measured by TEM (~170 nm) was slightly smaller than the DLS results. This discrepancy is likely attributed to nanoparticle hydration in the aqueous medium during DLS measurement.

X-ray diffractometry (XRD) confirmed the successful synthesis of the NPs (Fig. 1C). No novel peaks were discernible in MPB NPs, HMPB NPs, and Cu-HMPB NPs, suggesting that neither the hollow structure formation nor Mn^2+^/Cu^2+^ doping altered the original Prussian blue NP phase (standard: PDF No. 01–0239). We further employed X-ray photoelectron spectroscopy (XPS) to analyze the composition and valence states of Cu-HMPB@DSF/RSL3 NPs, demonstrating the co-existence of iron, copper, manganese, sulfur, chlorine, oxygen, and nitrogen within the Cu-HMPB@DSF/RSL3 NPs (Fig. 1D). The XPS survey spectrum revealed characteristic Cu^2+^ peaks at 944.1 and 963.1 eV. For the Cu 2p spectrum, peaks at 935.7 and 955.6 eV corresponded to Cu^2+^ (Cu 2p_3/2_ and Cu 2p_1/2_), while peaks at 933.1 and 952.7 eV were assigned to Cu⁺ bands (Fig. S5A). The Fe 2p spectrum exhibited three peaks at 707.3, 720.2, and 722.6 eV, corresponding to Fe^2+^ 2p_3/2_, Fe^2+^ 2p_1/2_, and Fe^3+^ 2p_1/2_, respectively (Fig. S5B). Peaks at 642.1 and 652.6 eV were attributed to Mn 2p_3/2_ and Mn 2p_1/2_ (Fig. S5C). In addition, the Mn 2p_3/2_ XPS peaks were divided into three well-defined peaks assigned to Mn^2+^ (639.9 eV), Mn^3+^ (642.1 eV), and Mn^4+^ (645.1 eV), respectively.

The zeta potentials of MPB NPs (−8.7 ± 1.2 mV), HMPB NPs (−2.8 ± 4 mV), and Cu-HMPB NPs (−15 ± 1 mV) were all negative (Fig. 1E). After adsorption of the cationic polymer PAH, the zeta potential of Cu-HMPB@DSF/PAH NPs markedly increased to 33 ± 0.7 mV, indicating successful surface modification. Subsequent loading of RSL3 onto Cu-HMPB@DSF/PAH NPs reduced the zeta potential to −18.5 ± 0.4 mV. Finally, the zeta potential of the fully assembled Membrane/Cu-HMPB@DSF/RSL3 NPs was −16.7 ± 0.3 mV, verifying the successful synthesis of the NPs.

 Nitrogen adsorption-desorption isotherm analysis demonstrated a Langmuir type IV profile for Cu-HMPB NPs, indicating mesoporous structure formation (Fig. 1F). Inductively coupled plasma-optical emission spectroscopy (ICP-OES) further verified the presence of Fe, Cu, Mn, and S in the NPs, confirming the successful encapsulation of DSF within the mesopores of Membrane/Cu-HMPB@DSF/RSL3 NPs (Table S1). The drug loading efficiency and encapsulation efficiency of DSF were determined to be 9.9 ± 0.5% (w/w) and 4.9 ± 0.2% (w/w), respectively. In addition, RSL3 loading was assessed by UV-Vis-NIR absorbance spectroscopy at 244 nm. The encapsulation efficiency of RSL3 was calculated to be 20.3%, with a corresponding drug loading of 10.1%. High-resolution TEM (HRTEM) elemental mapping revealed a uniform distribution of Fe, Mn, and Cu throughout the Cu-HMPB NP framework (Fig. 1G), demonstrating effective Mn^2+^/Cu^2+^ doping. X-ray fluorescence (XRF) analysis also confirmed the incorporation of Fe, Cu, Mn, Cl and S in Cu-HMPB@DSF/RSL3 NPs (Fig. 1H). Finally, SDS-PAGE demonstrated that Membrane/Cu-HMPB@DSF/RSL3 NPs exhibited a similar protein profile to C1498 AML cell membranes (Fig. 1I). In particular, membrane proteins specific to C1498 AML cells, including CD135 and CD47, were found on the NPs (Fig. 1J). These results collectively validate the successful synthesis of Membrane/Cu-HMPB@DSF/RSL3 NPs.

### Enzyme-mimetic catalysis

**Fig. 2:**
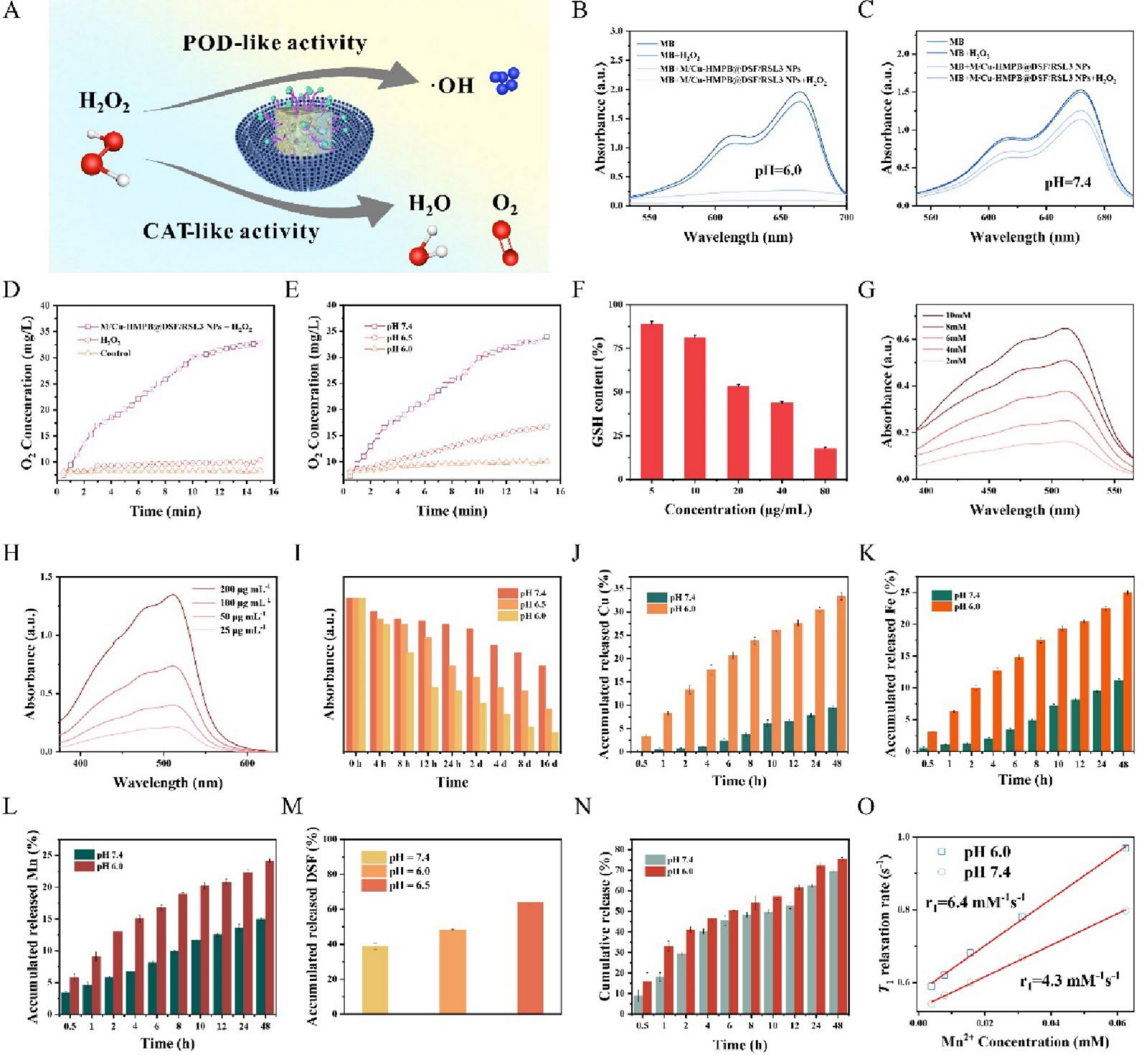
Catalytic activities and drug release performance of Membrane/Cu-HMPB@DSF/RSL3 NPs. (**A**) Schematic illustration of the peroxidase (POD)-like and catalase (CAT)-like catalytic mechanisms of Membrane/Cu-HMPB@DSF/RSL3 NPs. (**B**-**C**) UV-vis absorbance spectra of MB in (**B**) pH 6.0 and (**C**) pH 7.4 solutions with Membrane/Cu-HMPB@DSF/RSL3 NPs. (**D**) Oxygen generation under different conditions measured by a dissolved oxygen meter. (**E**) Oxygen generation of the NPs at varying pH values. (**F**) GSH depletion induced by Membrane/Cu-HMPB@DSF/RSL3 NPs. (**G**) Fe²⁺ concentration in the solution containing 200 µg/mL NPs with varying GSH concentrations. (**H**) Fe^2+^ concentration in the solution containing 20 mM GSH and varying NPs concentrations. (**I**) Absorbance-time profiles of NPs dissolved in SBF solutions (pH 7.4, 6.5, 6.0). (**J**) Cu, (**K**) Fe, and (**L**) Mn ion release from Membrane/Cu-HMPB@DSF/RSL3 NPs after dissolve in SBF solutions (pH 7.4, 6.0). (**M**) Cumulative DSF release profile over 48 h. (N) Drug RSL3 release profiles in SBF at pH 6.0 and 7.4. (O) *1/T₁* versus Mn^2+^ concentration plots

Encouraged by the observation that metal-containing nanoparticles exhibit peroxidase (POD)-like activity for hydroxyl radical (·OH) generation under acidic conditions (pH 6.0) and catalase (CAT)-like activity for oxygen (O_2_) production at neutral pH, [31] we investigated the enzyme-mimetic activities of the Membrane/Cu-HMPB@DSF/RSL3 NPs (Fig. 2A). To assess the POD-like activity of the NPs, methylene blue (MB) was used as a probe to detect ·OH generation. Under acidic conditions (pH 6.0), which simulate the mildly acidic cytoplasmic environment of AML cells, notable MB degradation was observed in the presence of the NPs (Fig. 2B). Correspondingly, the absorbance of MB at 665 nm decreased significantly over a 120-min period (Fig. S6). In contrast, minimal MB degradation was detected at pH 7.4, indicating that the POD-like activity of the NPs is negligible under neutral conditions (Fig. 2C). The generation of ·OH is likely mediated by the Fenton-like reaction catalyzed by Membrane/Cu-HMPB@DSF/RSL3 NPs. To evaluate the CAT-like activity of the NPs, dissolved O_2_ levels were measured in H_2_O_2_ solutions. A marked increase in O_2_ concentration was observed in the presence of both H_2_O_2_ and Membrane/Cu-HMPB@DSF/RSL3 NPs at pH 7.4 (Fig. 2D, Fig. S7) compared to H_2_O_2_ or PBS (control), confirming the ability of the nanoparticles to catalyze the decomposition of H_2_O_2_ into O_2_. Furthermore, the concentration of dissolved O_2_ increased progressively with rising pH (Fig. 2E).

Given the critical role of GSH in protecting cells from cuproptosis or ferroptosis, we next investigated whether the Membrane/Cu-HMPB@DSF/RSL3 NPs could directly deplete GSH. Using 5,5’-dithiobis (2-nitrobenzoic acid) (DTNB) as an indicator of GSH, we found that Membrane/Cu-HMPB@DSF/RSL3 NPs reduced GSH concentrations in a dose-dependent manner, with GSH concentrations decreasing to 17.63% of the control within 3 h (Fig. 2F). As GSH can be oxidized by Fe^3+^ released from the NPs, we further quantified the Fe^2+^ concentrations using 1,10-phenanthroline as an indicator. Indeed, UV-vis spectroscopy analysis revealed a concentration-dependent increase in 1,10-phenanthroline absorbance at 510 nm (Fig. 2G, H), indicating Fe^3+^- mediated GSH oxidation and the subsequent formation of Fe^2+^. Together, these results suggest that Membrane/Cu-HMPB@DSF/RSL3 NPs not only exhibit POD- and CAT-like enzymatic activities that promote ·OH and O_2_ generation, but also actively deplete intracellular GSH *via* direct redox reactions, thereby potentially intensifying ferroptosis or cuproptosis induction.

### Biodegradability

The AML cell-specific biodegradability of Membrane/Cu-HMPB@DSF/RSL3 NPs plays a pivotal role in achieving targeted delivery and controlled release of the metal ions and loaded drugs. We hypothesized that the bonding interactions between the doped Cu^2+^ ions and the -N ≡ C–Fe groups in the Membrane/Cu-HMPB@DSF/RSL3 NPs destabilize under mildly acidic conditions (Fig. S8), leading to pH-triggered degradation of the NPs within the acidic AML cytoplasm, enabling rapid release of metal ions (Fe, Cu, and Mn) and drug molecules (DSF and RSL3). More importantly, this degradation minimizes off-target release of metal ions and drug under neutral conditions, ensuring therapeutic specificity.

To evaluate this property, we assessed the pH-responsive biodegradation behavior of the NPs in simulated body fluid (SBF) at pH 7.4, 6.5, and 6.0, corresponding to physiological and AML-relevant acidic environments. The in vitro degradation behavior of Membrane/Cu-HMPB@DSF/RSL3 NPs was evaluated using a colorimetric assay, where the color of the NP solutions gradually faded over 16 days, with the most pronounced change observed at pH 6.0, suggesting significant spontaneous degradation. UV-vis spectroscopy analysis revealed a progressive decrease in absorbance at 725 nm across all three pH conditions, with the most significant reduction occurring at pH 6.0 (Fig. 2I, Fig. S9). The pH-dependent NP degradation was further confirmed using TEM imaging (Fig. S10).

We then quantified metal ion release using ICP-OES. Under neutral conditions (pH 7.4, 37 °C), 9.4% of copper ions were released from the NPs after 48 h. In contrast, exposure to mildly acidic conditions (pH 6.0) significantly accelerated copper release, achieving a cumulative release of 33.4% at 48 h (Fig. 2J). Similarly, the release of iron and manganese ions was also enhanced under acidic conditions, with cumulative release rates reaching 25.0% for Fe and 24.2% for Mn after 48 h (Fig. 2K, L).

Subsequently, we evaluated the release kinetics of DSF from the NPs under different pH conditions (Fig. 2M). At pH 7.4, only 38.9% of DSF was released after 48 h, whereas the cumulative release increased to 64.0% at pH 6.0. Given that DSF can rapidly react with Cu^2+^ ions to form copper-ethylenediamine tetraacetate (CU-ET) [32], a cytotoxic compound with a characteristic absorption peak at 400–500 nm, we quantified CU-ET generation using UV-vis spectroscopy. The absorbance intensity in this range was markedly higher at pH 6.0 compared to pH 7.4 (Fig. S11), indicating enhanced CU-ET formation under acidic conditions. In parallel, we analyzed the release kinetics of RSL3. The in vitro release profiles revealed that 75.4% of RSL3 was released at pH 6.0 within 48 h, compared to 69.6% at pH 7.4 (Fig. 2N).

Finally, we measured the magnetic properties of Membrane/Cu-HMPB@DSF/RSL3 NPs at different pH values (7.4 and 6.0) (Fig. S12). A concentration-dependent signal enhancement was observed in T1-weighted magnetic resonance (MRI) images. The T1 relaxivity of the NPs was determined to be 4.3 mM^− 1^ s^− 1^ (pH 7.4) and 6.4 mM^− 1^ s^− 1^ (pH 6.0) (Fig. 2O), suggesting enhanced Mn^2+^ release under mildly acidic conditions, which is consistent with the earlier ion release results and further supports the pH-responsive degradability of the NPs. These results collectively demonstrate that Membrane/Cu-HMPB@DSF/RSL3 NPs exhibit pH-responsive drug release behavior, with an accelerated release under mildly acidic conditions.

### Homotypic targeting of leukemia cells

**Fig. 3:**
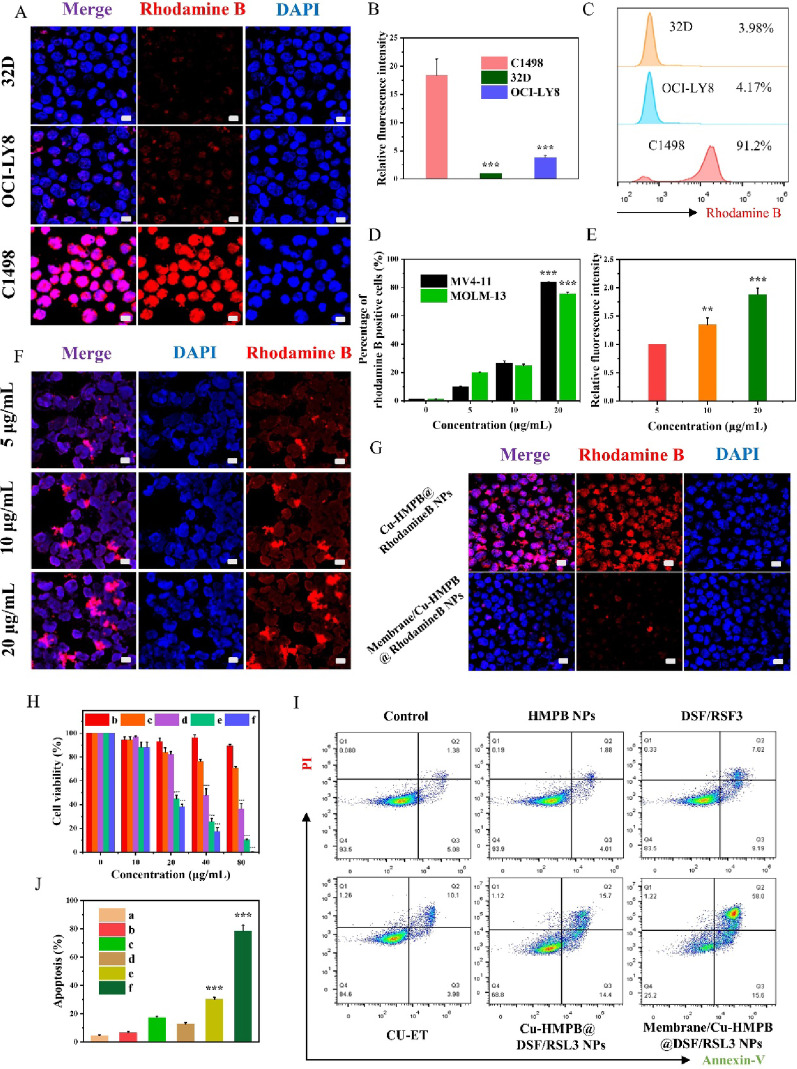
Cellular uptake and anti-AML effects of the Membrane/Cu-HMPB@DSF/RSL3 NPs. (**A**) Representative images 32D, OCI-LY8 and C1498 cells treated with Membrane/Cu-HMPB@RhodamineB NPs. Scale bars = 20 μm. (**B**) Relative fluorescence intensities of the cells in (**A**). (**C**) Flow cytometric analysis of the cells in (**A**). (**D**) Ratio of fluorescently positive MV4-11 and MOLM-13 cells treated with Membrane/Cu-HMPB@RhodamineB NPs measured by flow cytometry. (**E**) Dose-dependent uptake of Membrane/Cu-HMPB@Rhodamine B NPs in MOLM-13 cells, as measured by flow cytometry. (**F**) Fluorescent images of MOLM-13 cells treated with Membrane/Cu-HMPB@Rhodamine B NPs. (**G**) Representative images of RAW264.7 cells incubated with Cu-HMPB@DSF/RSL3 NPs (up) and Membrane/Cu-HMPB@DSF/RSL3 NPs (down). (**H**) Cell viability of C1498 cells treated with (a) PBS, (b) HMPB NPs, (c) DSF+RSL3, (d) CU-ET, (e) Cu-HMPB@DSF/RSL3 NPs, and (f) Membrane/Cu-HMPB@DSF/RSL3 NPs. (**I**) Flow cytometric analysis of cell apoptosis in (**H**). (**J**) Ratio of apoptotic cells in (**I**). Data are displayed as mean ± S.D. (*n* = 3). (N.S.: Not significant, * *P* < 0.05, ** *P* < 0.01, *** *P* < 0.001)

Having confirmed the enzyme-mimetic activities and pH-responsive degradability of Membrane/Cu-HMPB@DSF/RSL3 NPs, we next investigated their homotypic targeting capabilities against AML cells. We first prepared Membrane/Cu-HMPB@Rhodamine B NPs following the same synthetic pathway, with the drug molecules replaced by Rhodamine B to indicate the location of the NPs. Cellular uptake was assessed in three cell lines: C1498 (a mouse AML cell line), OCI-LY8 (a human diffuse large B-cell lymphoma cell line), and 32D (a non-tumor myeloblast-like cell line derived from mouse bone marrow). After 2 h of incubation, the fluorescence images showed strong signals in the C1498 cells but weak signals in the OCI-LY8 and 32D cells, suggesting that the C1498 cell membrane-coated NPs could be internalized by an identical cell type but not by other cells (Fig. 3A, B). Flow cytometry analysis further confirmed this selective uptake, showing 91.2% positivity in C1498 cells compared to ~4% in the other two lines (Fig. 3C). We further validated this effect in the human AML cell lines, MV4-11 and MOLM-13. Both the cell lines showed positive staining (Fig. 3D), whereas MOLM-13 cells demonstrated dose-dependent uptake of NPs (Fig. 3E, F). These findings collectively indicate that AML cell membrane-coated nanoparticles (NPs) can be selectively and effectively internalized by acute myeloid leukemia (AML) cells of both murine and human origin.

### Immune escape

In order to investigate the immune evasion characteristics of the nanoparticles (NPs), we administered Membrane/Cu-HMPB@RhodamineB NPs and non-coated Cu-HMPB@RhodamineB NPs to RAW264.7 mouse macrophages. Fluorescence imaging demonstrated a substantial cellular uptake of the non-coated NPs, whereas the Membrane/Cu-HMPB@Rhodamine B NPs exhibited minimal internalization (Fig. 3G). This observation suggests that non-coated NPs may be identified and eliminated by the immune system prior to drug delivery. In contrast, the NPs coated with leukemia cell membranes appear to evade immune detection, potentially through CD47-associated pathways. The capacity of Membrane/Cu-HMPB@Rhodamine B NPs to circumvent immune recognition and phagocytosis may enhance drug delivery efficacy and promote drug accumulation at the target site.

### Cytotoxicity

Subsequently, we investigated the cytotoxic effects of Membrane/Cu-HMPB@DSF/RSL3 NPs on acute myeloid leukemia (AML) cells. The CCK-8 assay was employed to evaluate cell viability in C1498 cells. Both Cu-HMPB@DSF/RSL3 NPs and Membrane/Cu-HMPB@DSF/RSL3 NPs significantly decreased cell viability in a dose-dependent manner, with the latter exhibiting the highest level of cytotoxicity. Importantly, the cytotoxic impact of Membrane/Cu-HMPB@DSF/RSL3 NPs was substantially greater than that observed with equivalent concentrations of free DSF+RSL3 or CU-ET (Fig. 3H). Flow cytometry analysis further demonstrated that Membrane/Cu-HMPB@DSF/RSL3 NPs induced the highest proportion of apoptotic cells compared to Cu-HMPB@DSF/RSL3 NPs and other treatments (Fig. 3I, J). To clarify whether NPs have the effect of jointly inducing ferroptosis and cuproptosis, We evaluated the cytotoxicity of Cu-HMPB@RSL3 NPs, Cu-HMPB@DSF NPs or Cu-HMPB@DSF/RSL3 NPs (0, 3.125, 6.25, 12.5, 25, 50 µg/mL) using the Cell Counting Kit-8 (CCK-8) assay, Herein, Cu-HMPB@DSF/RSL3 NPs exhibited a significant cytotoxic effect, And we calculate the ZIP synergy scores, indicating a synergistic cytotoxic effect (Fig. S13A, B). These findings indicate that Membrane/Cu-HMPB@DSF/RSL3 NPs exhibit potent cytotoxicity against AML cells.

### Membrane/Cu-HMPB@DSF/RSL3 NPs induce ferroptosis and cuproptosis in AML cells

**Fig. 4:**
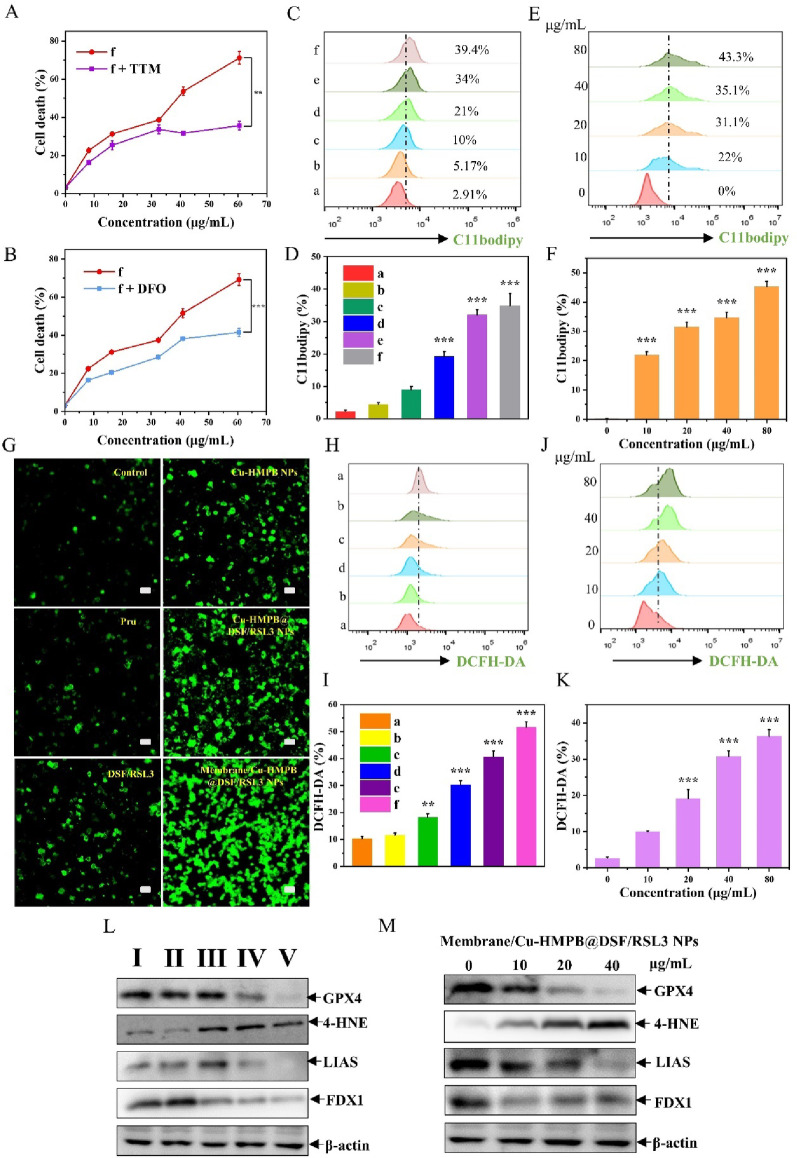
Membrane/Cu-HMPB@DSF/RSL3 NPs induce ferroptosis and cuprotosis in AML cells. (**A**) Viability of MOLM13 cells treated with Membrane/Cu-HMPB@DSF/RSL3 NPs alone or in combination with tetrathiomolybdate (TTM). (**B**) Viability of MOLM13 cells treated with Membrane/Cu-HMPB@DSF/RSL3 NPs alone or in combination with deferoxamine (DFO). (**C**) Flow cytometric analysis of MOLM-13 cells treated with (a) PBS, (b) HMPB NPs, (c) DSF+RSL3, (d) CU-ET, (e) Cu-HMPB@DSF/RSL3 NPs, and (f) Membrane/Cu-HMPB@DSF/RSL3 NPs using the C11-BODIPY assay. (**D**) Ratio of C11-BODIPY-positive cells in (**C**). (**E**) Levels of LPO in MOLM-13 cells treated with different concentrations of Membrane/Cu-HMPB@DSF/RSL3 NPs. (**F**) Ratio of C11bodipy-positive cells in (**E**). (**G**) Representative images of cells treated with the indicated reagents. The green fluorescence indicates intracellular ROS levels. Scale bars = 10 μm. (H) Flow cytometric analysis of MOLM-13 cells treated with different reagents using the DCFH-DA assay. (I) Ratio of DCFH-DA-positive cells in (**H**). (**J**) Levels of ROS in MOLM-13 cells treated with different concentrations of Membrane/Cu-HMPB@DSF/RSL3 NPs. (**K**) Ratio of DCFH-DA-positive cells in (**J**). (**L**) Expression levels of GPX4, 4-HNE, LIAS, and FDX1 proteins in MOLM-13 cells treated with (a) PBS, (b) HMPB NPs, (c) CU-ET, (d) Cu-HMPB@DSF/RSL3 NPs, and (e) Membrane/Cu-HMPB@DSF/RSL3 NPs. (**M**) Expression levels of GPX4, 4-HNE, LIAS, and FDX1 proteins in MOLM-13 cells treated with different concentrations of Membrane/Cu-HMPB@DSF/RSL3 NPs. Data are displayed as mean ± S.D. (*n* = 3). (N.S.: Not significant, * *P* < 0.05, ** *P* < 0.01, *** *P* < 0.001)

Following the confirmation of the cytotoxic effects of Membrane/Cu-HMPB@DSF/RSL3 NPs, we proceeded to examine the underlying therapeutic mechanisms. MOLM-13 cells were exposed to Membrane/Cu-HMPB@DSF/RSL3 NPs in the presence or absence of metal ion chelators: tetrathiomolybdate (TTM), a Cu^2+^ chelator, and deferoxamine (DFO), a Fe^3+^ chelator (Fig. 4A, B). Co-treatment with either TTM or DFO significantly restored cell viability, which was otherwise diminished by NP exposure. This observation suggests that the cytotoxicity of Membrane/Cu-HMPB@DSF/RSL3 NPs may be partially mediated through the release of iron and copper ions.

To explore ferroptosis as a potential mechanism, we evaluated intracellular lipid peroxidation (LPO) levels utilizing the C11-bodipy assay. In comparison to other treatment groups, Membrane/Cu-HMPB@DSF/RSL3 NPs markedly elevated LPO in MOLM-13 cells (Fig. 4C, D), exhibiting a dose-dependent relationship (Fig. 4E, F). Additionally, intracellular reactive oxygen species (ROS) levels were measured using 2’,7’-dichlorofluorescein diacetate (DCFH-DA). Consistent with the LPO findings, Membrane/Cu-HMPB@DSF/RSL3 NPs induced the highest ROS accumulation relative to Cu-HMPB@DSF/RSL3 NPs and other controls (Fig. 4G-I), also demonstrating a dose-dependent pattern (Fig. 4J, K).

Subsequently, we investigated the expression levels of proteins associated with cuproptosis and ferroptosis. The Membrane/Cu-HMPB@DSF/RSL3 NPs significantly downregulated the cuproptosis-related proteins LIAS and FDX1, as well as the ferroptosis-related protein GPX4 (Fig. 4L-M). Notably, the Membrane/Cu-HMPB@DSF/RSL3 NPs markedly upregulated the level of 4-HNE, a marker of lipid peroxidation (LPO).

These findings clearly indicate that the Membrane/Cu-HMPB@DSF/RSL3 NPs exert cytotoxic effects on acute myeloid leukemia (AML) cells by inducing both cuproptosis and ferroptosis (Fig. S14).

### Membrane/Cu-HMPB@DSF/RSL3 NPs inhibit AML in vivo

**Fig. 5:**
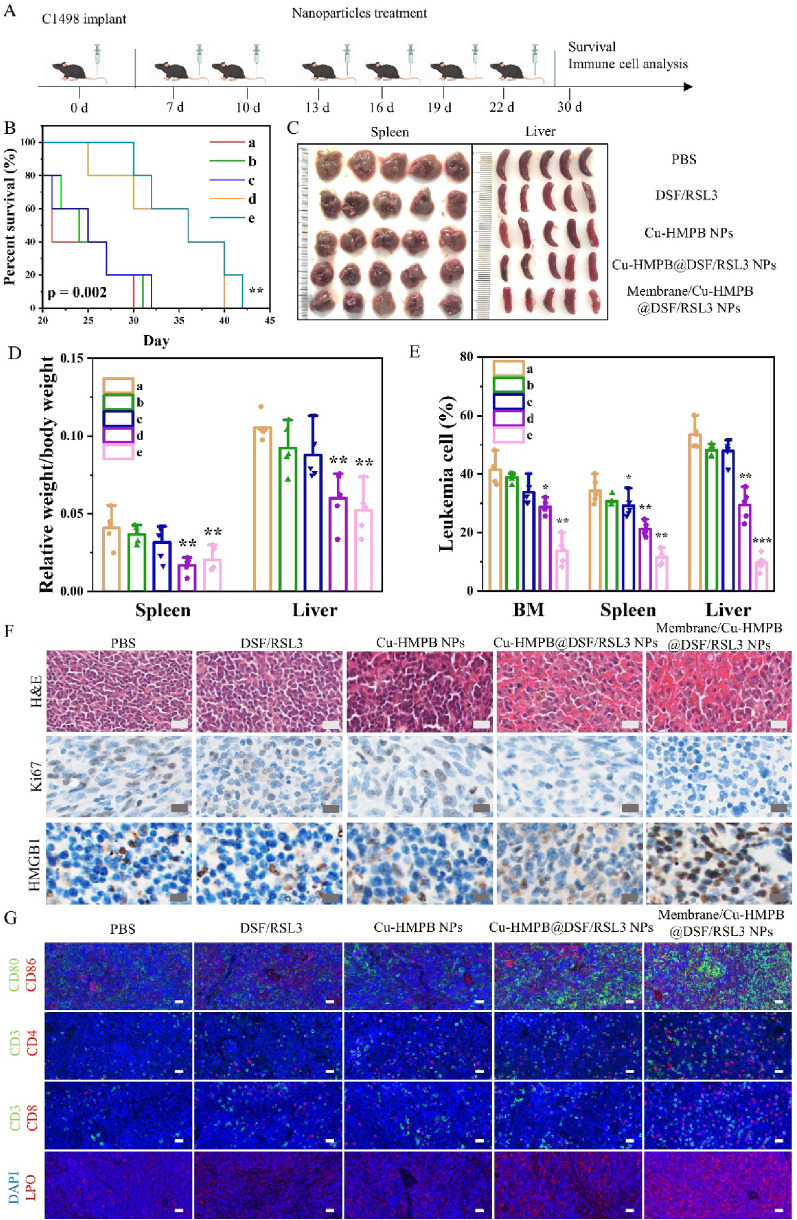
In vivo anti-leukemic effects of membrane/Cu-HMPB@DSF/RSL3 NPs in the C1498 mouse model (*n* = 5 per group). (**A**) Establishment and treatment of the C1498 mouse model. (**B**) Kaplan-Meier survival curves of the mice treated with (a) PBS, (b) DSF/RSL3, (c) Cu-HMPB, (d) Cu-HMPB@DSF/RSL3 NPs, and (e) membrane/Cu-HMPB@DSF/RSL3 NPs. (**C**) Representative images of spleens and livers harvested from treated mice. (**D**) Relative spleen and liver weights. (**E**) Proportions of GFP^+^ leukemic cells in bone marrow (BM), spleen, and liver. (**F**) H&E, Ki67 and HMGB1 staining of the spleen tissue. Scale bars = 20 μm. (**G**) Immunofluorescence images of the spleen tissue, showing mature dendritic cells (CD80^+^, CD86^+^), helper T cells (CD3^+^, CD4^+^), cytotoxic T lymphocytes (CD3^+^, CD8^+^), and intracellular lipid peroxidation (LPO) levels. Data are displayed as mean ± S.D. (*n* = 3). (N.S.: Not significant, * *P* < 0.05, ** *P* < 0.01, *** *P* < 0.001)

We conducted an in vivo evaluation of the anti-AML efficacy of Membrane/Cu-HMPB@DSF/RSL3 NPs. Initially, C1498-GFP+ cells were inoculated to establish murine models of acute myeloid leukemia (AML). On the seventh day post-inoculation, the mice were randomly allocated into five groups and received intravenous injections of either PBS, DSF/RSL3, Cu-HMPB NPs, Cu-HMPB@DSF/RSL3 NPs, or Membrane/Cu-HMPB@DSF/RSL3 NPs (Fig. 5A). In mice treated with Membrane/Cu-HMPB@DSF/RSL3 NPs, T1-weighted MRI revealed enhanced signals in the bone marrow, peaking at 8 h post-injection, indicating NP accumulation in the bone marrow (Fig. S15A, S15B). Survival analysis demonstrated that treatments with Cu-HMPB@DSF/RSL3 NPs and Membrane/Cu-HMPB@DSF/RSL3 NPs significantly improved survival compared to other treatments (Fig. 5B). Additionally, given that splenomegaly is a characteristic of leukemia, we evaluated the sizes and weights of the spleen and liver. Mice treated with Cu-HMPB@DSF/RSL3 NPs and Membrane/Cu-HMPB@DSF/RSL3 NPs exhibited reduced liver and spleen sizes and weights relative to other groups (Fig. 5C, D). The Membrane/Cu-HMPB@DSF/RSL3 NP group also showed the lowest number of GFP+ cells in the bone marrow, spleen, and liver (Fig. 5E). Immunohistochemical and H&E staining indicated that Membrane/Cu-HMPB@DSF/RSL3 NP treatment significantly decreased the number of leukemic cells in the spleen; and the number of Ki67-positive cells in the treatment group was markedly lower than in other groups (Fig. 5F). Immunohistochemical results also revealed that the high mobility group protein B1 (HMGB1) level in the Membrane/Cu-HMPB@DSF/RSL3 NP group was higher than that in the PBS group. Calreticulin (CRT) immunofluorescence analysis also corroborated these findings (Fig. S16). Compared with the PBS group, the Membrane/Cu-HMPB@DSF/RSL3 NP group exhibited elevated levels of adenosine triphosphate (ATP) and lactate dehydrogenase (LDH) (Fig. S17), indicating that leukemia cells underwent immunogenic cell death. Furthermore, compared to other treatments, Membrane/Cu-HMPB@DSF/RSL3 NP treatment resulted in a higher number of mature dendritic cells (CD80^+^, CD86^+^), helper T cells (CD3^+^, CD4^+^), and cytotoxic T lymphocytes (CD3^+^, CD8^+^) in the spleen, as corroborated by fluorescence intensity analysis (Fig. S18-S20). Consistent with the increased number of immune cells, western blotting revealed elevated expression levels of STING and phospho-TBK1 (p-TBK1) in leukemia cells following Membrane/Cu-HMPB@DSF/RSL3 NP treatment (Fig. S21), suggesting that released Mn^2+^ may activate the cGAS-STING pathway, thereby enhancing the immune response. Notably, increased levels of lipid peroxidation (LPO) were also observed in the spleens of the Membrane/Cu-HMPB@DSF/RSL3 NP group (Fig. 5G, Fig. S22), supporting the induction of ferroptosis in leukemic cells in vivo. The decreased levels of LIAS and FDX1 in the spleen treated with Membrane/Cu-HMPB@DSF/RSL3 NP indicate that this nanoparticle induces cuproptosis in leukemia cells in vivo (Fig. S23).

**Fig. 6:**
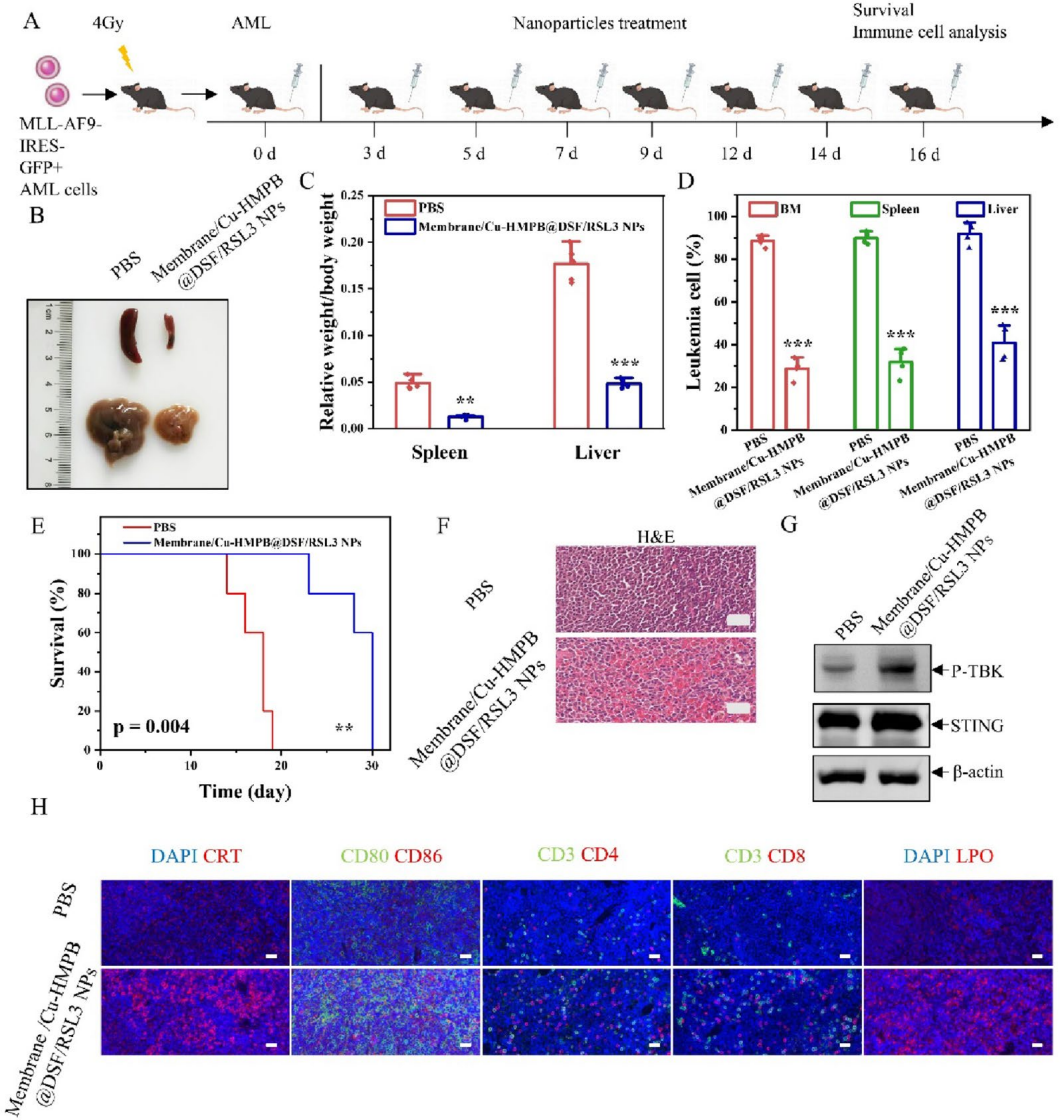
In vivo anti-leukemic effects of Membrane/Cu-HMPB@DSF/RSL3 NPs in the MLL-AF9 mouse model (*n* = 5 per group). (**A**) Establishment and treatment of the MLL-AF9 mouse model. (**B**) Representative images of spleens and livers harvested from treated mice. (**C**) Relative spleen and liver weights. (**D**) Proportions of GFP^+^ leukemic cells in bone marrow (BM), spleen, and liver. (**E**) Kaplan-Meier survival curves of the mice treated with PBS or Membrane/Cu-HMPB@DSF/RSL3 NPs. (**F**) H&E staining of the spleen tissue. Scale bars = 20 μm. (**G**) Expression levels of STING and p-TBK1 in spleen tissue. (**H**) Immunofluorescence images of the spleen tissue, showing CRT, mature dendritic cells (CD80^+^, CD86^+^), helper T cells (CD3^+^, CD4^+^), cytotoxic T lymphocytes (CD3^+^, CD8^+^), and intracellular lipid peroxidation (LPO) levels. Scale bars = 20 μm. Data are displayed as mean ± S.D. (*n* = 3). (N.S.: not significant, * *P* < 0.05, ** *P* < 0.01, *** *P* < 0.001)

To further substantiate the anti-AML efficacy of Membrane/Cu-HMPB@DSF/RSL3 NPs, an additional in vivo AML model driven by the MLL-AF9 fusion oncogene was employed. Mice were intravenously injected with MLL-AF9-IRES-GFP⁺ leukemic cells to establish the disease model and subsequently treated with either PBS or Membrane/Cu-HMPB@DSF/RSL3 NPs (Fig. 6A). Consistent with the observations in the C1498 AML model, mice treated with Membrane/Cu-HMPB@DSF/RSL3 NPs exhibited significantly reduced liver and spleen sizes and weights compared to the PBS group (Fig. 6B, C). Flow cytometry analysis revealed markedly lower numbers of GFP⁺ leukemic cells in the bone marrow, spleen, and liver (Fig. 6D), along with significantly prolonged survival (Fig. 6E). Histological evaluation via H&E staining confirmed decreased leukemic infiltration in the spleen following NP treatment (Fig. 6F). Western blotting again confirmed the upregulation of STING and p-TBK1 in the Membrane/Cu-HMPB@DSF/RSL3 NP-treated group (Fig. 6G), supporting the role of Mn²⁺-mediated cGAS-STING pathway activation in promoting immune responses. Furthermore, immunofluorescence analysis demonstrated increased numbers of CRT, mature dendritic cells, helper T cells, and cytotoxic T lymphocytes in the spleen, as well as elevated LPO levels (Fig. 6H) and decreased LIAS and FDX1 levels (Fig. S23, S24), suggesting the simultaneous activation of anti-tumor immunity, cuproptosis and ferroptosis. Together with the findings from the C1498 model, these results demonstrate the broad in vivo anti-AML efficacy of Membrane/Cu-HMPB@DSF/RSL3 NPs across genetically distinct AML subtypes, likely through the dual induction of ferroptosis and immune activation.

### **Membrane/Cu-HMPB@DSF/RSL3 NPs promote immune response** in vivo

**Fig. 7:**
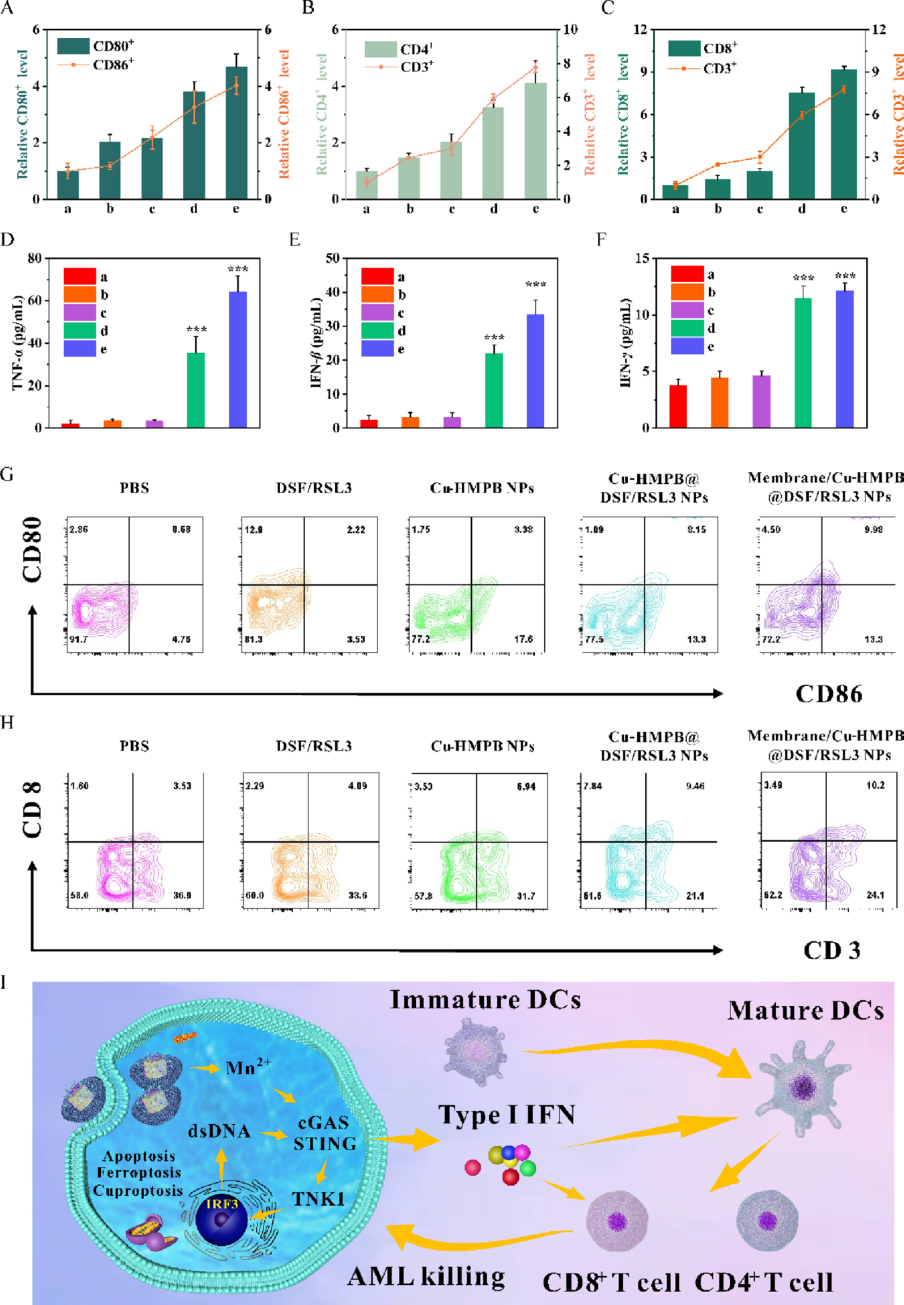
Membrane/Cu-HMPB@DSF/RSL3 NPs enhance anti-tumor immunity in the C1498 AML mouse model. (**A**-**C**) Quantification of CD80^+^/CD86^+^ (**A**), CD3^+^/CD4^+^ (**B**), and CD3^+^/CD8^+^ (**C**) in the bone marrow. (**D**-**F**) ELISA quantification of serum cytokine levels, including TNF-α (**D**), IFN-*β* (**E**), and IFN-*γ* (**F**), in different treatment groups. (**G**-**H**) Flow cytometric analysis of CD80⁺/CD86⁺ dendritic cells (**G**) and CD3⁺/CD8⁺ cytotoxic T cells (**H**) in the bone marrow of membrane/Cu-HMPB@DSF/RSL3 NP-treated mice. Data are displayed as mean ± S.D. (*n* = 3). (N.S.: Not significant, * *P* < 0.05, ** *P* < 0.01, *** *P* < 0.001). (**I**) Schematic diagram of immunotherapy mechanisms

Given that Membrane/Cu-HMPB@DSF/RSL3 NP-treatment increased immune cell infiltration in the bone marrow, we further quantified immune cell populations in the bone marrow and measured serum cytokine levels in C1498 mice. Compared with other treatment groups, Membrane/Cu-HMPB@DSF/RSL3 NP led to the highest amount of CD80^+^/CD86^+^ (Fig. 7A), CD3^+^/CD4^+^ (Fig. 7B), and CD3^+^/CD8^+^ (Fig. 7C) in the bone marrow sample. In parallel, ELISA revealed significantly elevated concentrations of proinflammatory and immunostimulatory cytokines, including TNF-α, IFN-*β*, IFN-*γ*, and IL-6, in the serum of treated mice (Fig. 7D-F, Fig. S25). Flow cytometric analysis further confirmed the increase of CD80^+^/CD86^+^ dendritic cells (Fig. 7G) and CD3^+^/CD8^+^ cytotoxic T cells (Fig. 7H) in the bone marrow following treatment. Notably, a similar increase in CD3^+^/CD8^+^ cytotoxic T lymphocytes was also observed in the bone marrow of MLL-AF9 AML model mice treated with Membrane/Cu-HMPB@DSF/RSL3 NPs (Fig. S26), supporting the generalizability of the immunostimulatory effect across AML subtypes. Membrane/Cu-HMPB@DSF/RSL3 NPs synergistically treat AML through apoptosis, ferroptosis and cuproptosis, inducing immunogenic cell death (ICD) in AML cells. The released type I IFNs (IFN-*β*) promote dendritic cells maturation, and matured dendritic cells activate cytotoxic T lymphocytes, triggering anti-leukemic immune responses. Mn²⁺ released by the NPs enhance the cGAS-STING pathway, thereby improving the efficacy of metalloimmunotherapy against AML. (Fig. 7I).

### Biosafety of membrane/Cu-HMPB@DSF/RSL3 NPs

To evaluate the biosafety of Membrane/Cu-HMPB@DSF/RSL3 NPs, we performed serum biochemical analyses in C1498 AML-bearing mice 30 days after the beginning of the treatment. The levels of alanine aminotransferase (ALT), blood urea nitrogen (BUN), creatinine (CRE), and aspartate aminotransferase (AST) remained within normal physiological ranges (Fig. S27), indicating no significant hepatic or renal toxicity. Hemocompatibility was further assessed via a standard hemolysis assay. Erythrocytes treated with Membrane/Cu-HMPB@DSF/RSL3 NPs at concentrations below 600 µg/mL exhibited minimal hemolysis (< 5%), with a measured hemolysis rate of 4.57% ± 0.89%, confirming good blood compatibility (Fig. S28). Finally, histological examination of major organs, including the spleen, lungs, heart, and kidneys, revealed no pathological abnormalities in either the NP-treated or control (saline-treated) groups (Fig. S29), further supporting the in vivo safety and non-toxicity of Membrane/Cu-HMPB@DSF/RSL3 NPs.

## Discussion

In this study, we developed a multifunctional, pH-responsive, and degradable nanoparticle system, Membrane/Cu-HMPB@DSF/RSL3 NPs, for the treatment of AML. By leveraging the structural tunability of Prussian blue-based frameworks and the homotypic targeting conferred by leukemia cell membrane coating, this platform enables efficient and selective delivery of copper, iron, and manganese ions, along with two small-molecule drugs (DSF and RSL3), particularly under the mildly acidic conditions of AML cells. The nanoparticles exhibit POD- and CAT-like enzyme-mimetic activities to promote ·OH and O_2_ generation and deplete intracellular GSH, thereby amplifying ferroptosis and cuproptosis triggered by the released iron and copper ions. Simultaneously, the manganese ions activate the innate immune system by stimulating the cGAS-STING pathway, leading to the recruitment of immune cells that facilitate AML eradication. We demonstrated the potent anti-AML efficacy of this nanoplatform in both in vitro and in vivo models, while confirming minimal systemic toxicity. Collectively, this study presents a novel and synergistic therapeutic strategy for AML that integrates targeted delivery, redox-mediated cell death, and immune activation, addressing the limitations of current monotherapies and highlighting the value of immunogenic nanotherapies.

Ferroptosis and cuproptosis are two distinct non-apoptotic modes of programmed cell death, both dependent on redox-active metal ions and characterized by impaired oxidative stress responses. Our nanoparticle system exploits these vulnerabilities by releasing excess iron and copper ions, which catalyze Fenton-like reactions and amplify LPO. The incorporated small-molecule drug DSF serves as an ionophore of copper and promotes the formation of the cytotoxic CU-ET complex [33], intensifying redox stress and mitochondrial dysfunction. Meanwhile, RSL3 inhibits GPX4, a key antioxidant enzyme that prevents ferroptosis, thereby sensitizing AML cells to oxidative damage. Notably, the intrinsic GSH-scavenging capability of our nanoparticles further weakens intracellular antioxidant defenses, forming a self-reinforcing cycle of redox collapse. The essential role of metal ion release was further verified by the partial reversal of cytotoxicity upon treatment with DFO (an iron chelator) and TTM (a copper chelator), indicating that both ferroptosis and cuproptosis are critically involved. This coordinated activation of ferroptosis and cuproptosis was confirmed by chelator rescue assays and elevated ROS/LPO levels, underscoring the promise of simultaneously engaging multiple regulated cell death pathways in AML therapy.

Another highlight of our design is the incorporation of manganese ions, which further enhances the therapeutic efficacy of our system through immune activation. Mn^2+^ is known to be a critical activator of the cGAS-STING pathway, a critical component of innate immunity. The activation of the cGAS-STING pathway leads to increased expression of type I interferons and pro-inflammatory cytokines, promoting the maturation of dendritic cells and recruitment of cytotoxic T lymphocytes into the bone marrow. In our in vivo experiments, Membrane/Cu-HMPB@DSF/RSL3 NP-treated mice showed elevated expression of STING and p-TBK1, alongside increased numbers of CD8⁺ T cells, dendritic cells, and helper T cells in both spleen and bone marrow. Notably, the activation of the cGAS-STING pathway by Mn^2+^ not only stimulates innate immune responses but also contributes to the promotion of ferroptosis, thereby enhancing the overall anti-leukemic effect [34]. This crosstalk between immune activation and redox-mediated cytotoxicity positions Mn^2+^ as a dual-function element within our nanoparticle design, serving both as an immunostimulant and as a ferroptosis sensitizer.

In addition to biochemical and immunological modulation, the homotypic targeting capability of the membrane-coated nanoparticles provides a crucial advantage in enhancing targeted delivery. This strategy, which involves cloaking nanoparticles with membranes derived from the source cancer cells, has been successfully applied in various nanoplatforms to achieve immune evasion and homotypic recognition [35–38]. Interestingly, despite the use of murine AML cell membranes in our study, the homotypic targeting effect was preserved in both murine and human AML cells. This may be explained by the conservation of key membrane proteins across species, as well as the generalized capacity of leukemia cells to engage in adhesive interactions with each other within the hematopoietic microenvironment.

Beyond its demonstrated efficacy in AML, our Prussian blue-based nanoplatform also holds substantial promise for broader oncological applications due to its structural versatility and modular design. The porous framework of Membrane/Cu-HMPB allows for facile substitution or co-loading of alternative therapeutic ions or other small-molecule drugs tailored to the vulnerabilities of different tumor types. Additionally, the membrane-coating strategy can be adapted using patient-derived cancer cell membranes or engineered membrane mimetics to achieve tumor-specific targeting in solid or hematological malignancies. Such adaptability not only enhances the translational potential of this system but also opens new avenues for developing personalized nanotherapeutics that integrate redox regulation, immune activation, and targeted delivery. Future studies exploring these modifications may further expand the therapeutic scope and clinical relevance of this platform in precision oncology.

## Conclusion

In summary, we developed a multifunctional, pH-responsive, and degradable nanoparticle system (Membrane/Cu-HMPB@DSF/RSL3 NPs) that simultaneously induces ferroptosis and cuproptosis while activating immune responses in AML. In Membrane/Cu-HMPB@DSF/RSL3 NPs, DSF and copper ions induce cuproptosis in leukemia cells, while RSL3 induces ferroptosis. Iron ions deplete GSH, indirectly promoting both ferroptosis and cuproptosis. Manganese ions, on the other hand, activate the cGAS-STING pathway in anti-leukemia immune responses.

We acknowledge the complexity of this system and find it difficult to determine which type of death mode dominates. From our results, the therapeutic effects of the simple DSF combined with RSL3 drugs, as well as the effect of the Prussian blue material loaded with iron ions, were all weaker than that of the final nanomedicine platform. This suggests that the copper ions and manganese ions that are loaded may significantly contribute to the anti-leukemia effect, and the manganese ions mediating immunogenic therapy is also an important part of the combined effect. Therefore, we preliminarily conclude that in the entire anti-leukemia effect, there exists a combined effect of copper death, iron death, cell apoptosis, and immunogenic death.

This strategy significantly reduced leukemia burden, activated immune response, and prolonged survival in vivo, offering a promising and adaptable approach for AML therapy. This multifunctional nano-cloaking platform offers a promising strategy for treating hematological malignancies and warrants further clinical investigation.

## Materials and methods

### Materials

Ferricyanide (K_3_[Fe(CN)_6_]_3_·H_2_O), cupric acetate monohydrate (Cu(CH_3_COOH)_2_·H_2_O), and poly(vinylpyrrolidone) (PVP, K30) were purchased from Ponsure Biological Co., Ltd (Shanghai, China); Disulfiram (DSF), poly(allylamine hydrochloride) (PAH), trisodium citrate dihydrate, ethanol, Phosphate buffer solution (PBS), and simulated body fluid (SBF) were obtained from Sigma-Aldrich Co. (Shanghai, China); sodium hexacyanoferrate (II) decahydrate (99%), manganese (II) chloride tetrahydrate (99%) and iron (III) chloride hexahydrate (97%) were purchased from Sigma-Aldrich (USA). 4, 6-diamidino-2-phenylindole (DAPI) were obtained from Molecular Probes Inc. (USA). The RSL3 was obtained from Baiolebo Biotechnology Co., Ltd. (Beijing, China). Antibodies were sourced as follows: anti-HMGB1 (GB11103-100, Servicebic, China), anti-calreticulin (ab92516, Abcam, UK), anti-CD3 (78588, Cell Signaling Technology, USA), anti-CD8 (ab217344, Abcam, UK), and anti-CD4 (ab183685, Abcam, UK), anti-CD80 (254579, Abcam, UK), anti-CD86 (19589, Cell Signaling Technology, USA), Ki67 (12202, Cell Signaling Technology, USA), and anti-LPO (115521, Invitrogen, USA). GSH and GSSG assay kit (S0053, Beyotime, China). TUNEL cell apoptosis detection kit (G1502-100T) were purchased from (Servicebic, China). The other chemicals were of analytical grade and used without further purification. Other chemicals were supplied by J&K Scientific Co., Ltd. (Beijing, China) and Ponsure Biotechnology Co., Ltd. (Shanghai, China).

###  Characterization techniques

 Transmission electron microscopy (TEM) images were acquired on a JEM-2100 instrument (JEOL, Japan) operated at 200 kV. X-ray powder diffraction (XRD) was performed using a SAXSessmc2 instrument (Anton Paar) equipped with Cu Kα radiation (45 kV, 40 mA, 2θ range 3–90°). X-ray photoelectron spectroscopy (XPS) was conducted using a ThermoFisher Escalab 250Xi instrument with Al Kα radiation. Specific surface area and pore diameter were analyzed *via* classical adsorption methods. A 100 mg sample was vacuum-degassed at 473.15 K for 20 h, and the adsorption-desorption isotherm was recorded on a fully automated gas adsorption instrument (Autosorb-iQ, Anton Paar). Pore size distribution was calculated using the Barrett-Joyner-Halenda (BJH) model, while the BET model determined the specific surface area. Fourier transform infrared spectroscopy (FTIR) was performed on an AVATAR-330 spectrometer (Thermo Nicolet, USA), and UV-vis spectra were obtained using a UV-3600 spectrophotometer (Shimadzu, Japan). Nanoparticle dispersions (1 mg/mL) in aqueous media were prepared *via* ultrasonication prior to hydrodynamic diameter and zeta potential measurements on a Nano-ZS instrument (Malvern, UK). Flow cytometry was performed using a FACS Calibur cytometer (BD Biosciences, USA), and fluorescence imaging was conducted on an LSM800 confocal laser scanning microscope (Carl Zeiss, Germany). Elemental analysis was carried out *via* inductively coupled plasma-optical emission spectroscopy (ICP-OES, Agilent Technologies 5110, USA).

## Experimental section

### Synthesis of Mn^2+^-containing prussian blue nanoparticles (MPB NPs)

Na_4_[Fe(CN)_6_]·10H_2_O (34.18 mg) was dissolved in 10 mL ultra-pure water, and FeCl_3_·6H_2_O (9.72 mg) and MnCl_2_·4H_2_O (8.7 mg) were dissolved in another 10 mL ultra-pure water. A 20 mL aqueous solution of Na_4_[Fe(CN)_6_]·10H_2_O (34.18 mg) and a mixed solution of FeCl_3_·6H_2_O and MnCl_2_·4H_2_O (20 mL) were simultaneously added at a rate of 2 mL h⁻¹ into 200 mL of ultra-pure water under constant stirring using a syringe pump. The mixture was stirred for 1 h and then centrifuged at 11 000 rpm (15 000 × g) for 15 min. The MPB NPs were washed three times with ultra-pure water and ethanol, and dried under vacuum.

### Synthesis of HMPB NPs

 The MPB NPs were transformed into PVP-modified hollow mesoporous Prussian blue (HMPB NPs) through a controlled chemical etching process. Specifically, MPB NPs (10 mg) and PVP (50 mg) were dispersed in 10 mL of 1 M HCl solution in a Teflon vial and stirred at room temperature for 4 h. The reaction mixture was then transferred to a stainless steel autoclave and heated at 140 ℃ for 3 h under controlled conditions. After cooling to room temperature, the resulting HMPB NPs were collected *via* three rounds of centrifugation at 11 000 rpm (15 000 × g) for 20 min each and washed three times with ethanol and ultra-pure water to remove unreacted reagents.

### Synthesis of Cu-HMPB NPs

Copper ions were incorporated into the skeleton of HMPB NPs *via* an ion-exchange method. Specifically, the synthesized HMPB NPs (25 mg) were dispersed in distilled water (10 mL) with Na_3_C_6_H_5_O_7_·2H_2_O (67.5 mg), Cu(CH_3_COO)_2_·H_2_O (22 mg), and PVP (250 mg). The mixture was stirred at room temperature for 3 h. Subsequently, K_3_[Fe(CN)_6_] (33 mg) was introduced, and the reaction was continued under stirring for an additional 2 h. After aging for 24 h at room temperature, the Cu-HMPB NPs were collected via three rounds of centrifugation at 11 000 rpm (15 000 × g) for 20 min each and washed three times with ethanol and ultra-pure water to remove unreacted reagents.

### Synthesis of Cu-HMPB@DSF NPs

DSF (40 mg) was dissolved in chloroform (2 mL) and the solution was stirred for 10 min at room temperature. Subsequently, Cu-HMPB NPs (20 mg) were added to the DSF solution, and the mixture was stirred continuously for 12 h under ambient conditions. The Cu-HMPB@DSF NPs were collected via centrifugation at 11 000 rpm (15 000 × g) for 15 min and washed three times with ethanol and deionized water to remove unreacted DSF and residual chloroform.

### Synthesis of Cu**-**HMPB@DSF/PAH NPs

 The Cu-HMPB@DSF (10 mg) was dispersed in 2.5 mL of PAH solution (5 mg mL⁻¹) and stirred continuously for 1 h under ambient conditions. Subsequently, the Cu-HMPB@DSF/PAH NPs were collected via centrifugation at 11 000 rpm (15 000 × g) for 15 min and washed three times with deionized water to remove unreacted PAH.

### Synthesis of Cu-HMPB@DSF/RSL3 NPs

Hyaluronic acid (HA, 5 mg) was dispersed in ultrapure water (5 mL) and sonicated for 30 min. Next, 1-ethyl-3-(3-dimethylaminopropyl)carbodiimide (EDC, 5 mg) and N-hydroxysuccinimide (NHS, 2.5 mg) were added to activate the carboxyl groups of HA. The mixture was stirred for 1 h at room temperature. Then, 10 mg of Cu-HMPB@DSF/PAH NPs were introduced into the activated HA solution. The resulting mixture was probe-sonicated (30% power, 15 s intervals) for 15 min to ensure homogeneity.

For RSL3 loading, 5 mg of RSL3 was dissolved in DMSO (10 mL) and diluted with deionized water (2 mL). The DMSO solution was added to the HA-functionalized NPs mixture and stirred for 12 h. The product was collected *via* centrifugation at 11 000 rpm (15 000 × g) for 15 min and washed three times with deionized water. The self-assembly process on the NPs surface yielded Cu-HMPB@DSF/RSL3 NPs with controlled drug loading.

### Synthesis of** m**embrane**-**functionalized NPs (membrane/Cu-HMPB@DSF/RSL3 NPs)

Membrane/Cu-HMPB@DSF/RSL3 NPs were prepared by mixing 1 mL of Cu-HMPB@DSF/RSL3 NPs (1 mg mL⁻^1^) with 1 mL of C1498 acute myeloid leukemia (AML) cell membranes extracted *via* enzymatic digestion from mouse bone marrow. The mixture was sonicated for 5 min in an ultrasonic bath (100 W, 40 kHz) to homogenize the components. Next, the mixture was extruded 10 times through a 200 nm pore-size polycarbonate membrane using a LiposoFast-Basic extruder (Avanti Polar Lipids). The final product was centrifuged at 11 000 rpm (15 000 × g) for 15 min to remove unbound membranes.

### Evaluation of biodegradability of membrane/Cu-HMPB@DSF/RSL3 NPs

Membrane/Cu-HMPB@DSF/RSL3 NPs (30 mg) were dispersed in SBF (30 mL) at pH 7.4, 6.5, or 6.0. The solution was stirred continuously at 37 °C to mimic physiological conditions. Aliquots (2 mL) were collected at predetermined time intervals (0 h, 4 h, 8 h, 12 h, 24 h; 2 days, 4 days, 8 days, and 16 days) for UV-vis spectrophotometric analysis (λ = 510 nm).

Ion release experiments were performed using procedures analogous to those described above. The concentrations of Mn, Fe, and Cu ions released from Membrane/Cu-HMPB@DSF/RSL3 NPs were quantified by ICP-OES (Agilent Technologies 5110, USA). Briefly, 10 mg of Membrane/Cu-HMPB@DSF/RSL3 NPs NPs were dispersed in SBF (30 mL) at pH 7.4 or 6.0 and vigorous stirring at 37 °C. The mixture was centrifuged at 11 000 rpm (15 000 × g) for 15 min, and supernatants were collected. Subsequently, the concentrations of Fe, Cu, and Mn in the release media were determined *via* ICP-OES at predetermined time intervals (0, 1, 2, 4, 5, 6, 8, 10, 12, 24, and 48 h).

In a third set of experiments, a 200 µg mL⁻¹ suspension of Membrane/Cu-HMPB@DSF/RSL3 NPs was prepared in 10 mL of deionized water and incubated with GSH (2, 4, 6, 8, or 10 mM) under dark conditions at 37 °C for 12 h. Subsequently, 0.1 mg mL⁻¹ aqueous 1,10-phenanthroline was added to the reaction mixture. The absorbance of the resultant solution was measured by UV-vis spectrophotometry (λ = 510 nm), corresponding to the formation of Fe^2+^-phenanthroline complexes.

To investigate the effect of NP concentration, Membrane/Cu-HMPB@DSF/RSL3 NPs at varying concentrations (25–200 µg mL⁻¹) were combined with 20 mM GSH and incubated under identical conditions. After reaction completion, 0.1 mg mL⁻¹ 1,10-phenanthroline was introduced, and the absorbance was quantified at 510 nm.

### Evaluation of drug loading and release behavior

 The drug loading (DL) and encapsulation efficiency (EE) of disulfiram (DSF) were quantified using ICP-OES (Agilent Technologies 5110, USA). Briefly, Membrane/Cu-HMPB@DSF/RSL3 NPs (10 mg) were digested in 200 µL of aqua regia (3:1 HCl: HNO_3_) at 80 °C for 12 h to ensure complete decomposition. the resulting solution was diluted to 10 mL with deionized (DI) water. Sulfur content in the NPs was analyzed via ICP-OES under optimized conditions (RF power: 1150 W, nebulizer gas flow: 0.8 L/min).

In the in vitro drug release experiment, 30 mg of Membrane/Cu-HMPB@DSF/RSL3 NPs were dispersed in 30 mL of SBF buffers at pH 7.4 or 6.0. The solution was shaken vigorously at 200 rpm under dark conditions at 37 °C for 48 h. The mixture was centrifuged at 11,000 rpm (15,000 × g) for 15 min, and the supernatants were collected. The extent of drug release in the supernatant was quantified by ICP-OES under optimized conditions.

The drug loading (DL) and encapsulation efficiency (EE) of RSL3 were determined using a UV-Vis-NIR spectrophotometer (Agilent Cary 60, USA) via measuring absorbance at 244 nm. Unbound RSL3 in the supernatant was quantified by analyzing the absorbance of the solution at the same wavelength. The DL and EE of RSL3 were calculated using the following equations:

$$\mathrm{D}\mathrm{r}\mathrm{u}\mathrm{g}\,\mathrm{l}\mathrm{o}\mathrm{a}\mathrm{d}\mathrm{i}\mathrm{n}\mathrm{g}\mathrm{\%}=\frac{\mathrm{M}\mathrm{a}\mathrm{s}\mathrm{s}\text\,{o}\mathrm{f}\,\mathrm{d}\mathrm{r}\mathrm{u}\mathrm{g}\,\mathrm{i}\mathrm{n}\,\mathrm{N}\mathrm{P}\mathrm{s}}{\mathrm{T}\mathrm{o}\mathrm{t}\mathrm{a}\mathrm{l}\,\mathrm{m}\mathrm{a}\mathrm{s}\mathrm{s}\,\mathrm{o}\mathrm{f}\,\mathrm{N}\mathrm{P}\mathrm{s}}\times100\mathrm{\%}$$

$$\begin{array}{l}{\rm{Encapsulation}}\, {\rm{efficiency}}\% \\ = \frac{{{\rm{Mass}}\, {\rm{of}} {\rm{drug}} {\rm{in}} NPs}}{{{\rm{Total}} \,{\rm{mass}} \,{\rm{of}}\, {\rm{drug}}}} \times 100\% \end{array}$$

To investigate the RSL3 release behavior, 1 mg of Membrane/Cu-HMPB@DSF/RSL3 NPs was dispersed in 5 mL of SBF (pH 7.4 or 6.0). The solutions were transferred into dialysis bags (MWCO = 10,000 Da) and sealed. Subsequently, the dialysis bags were immersed in 40 mL of SBF (pH 7.4 or 6.0) and placed in a thermostatic shaker incubator at 200 rpm and 37 °C. At predetermined time intervals (0.5, 1, 2, 4, 6, 8, 10, 12, and 24 h), 3 mL of the release medium was collected. The concentration of RSL3 was quantified using UV-Vis-NIR spectrophotometry (Agilent Cary 60, USA) at 244 nm. All experiments were performed in triplicate, and results are expressed as mean ± standard deviation (SD).

### Evaluation of in vitro peroxidase-like activity assay

To investigate the peroxidase-like activity of Membrane/Cu-HMPB@DSF/RSL3 NPs, 400 µg mL⁻¹ NPs and 5 mM GSH were dissolved in 10 µg mL⁻¹ methylene blue (MB) solution (PBS buffer, pH 7.4 or 6.0). Next, 100 mM H_2_O_2_ was added to initiate the reaction. The mixture was stirred at 200 rpm under dark conditions at 37 °C for 2 h. The absorbance of MB at 665 nm was quantified using UV-vis spectrophotometry.

### Evaluation of in vitro catalase-mimetic activity

To assess the catalase-like activity of Membrane/Cu-HMPB@DSF/RSL3 NPs, 0.5 mg of NPs and H_2_O_2_ (final concentration 5% (v/v)) were suspended in 10 mL PBS buffer (10 mM, pH 7.4, 6.5, or 6.0). The reaction mixture was transferred to a quartz cuvette and continuously stirred at 200 rpm under dark conditions at 37 °C. Dissolved-oxygen levels were monitored in real-time using a dissolved-oxygen meter (Shanghai REX, JPBJ-608, China).

### Evaluation of GSH depletion capacity

To assess the GSH depletion induced by Membrane/Cu-HMPB@DSF/RSL3 NPs, 25–200 µg/mL NPs and GSH (1 mM) were co-incubated in PBS buffer (10 mM, pH 6.0) at 37 °C for 12 h. DTNB (1 mg/mL, final concentration 10 µM) was added to the mixture for 30 min. The absorbance was measured at 412 nm using a UV-vis spectrophotometer.

### Cell uptake of the nanoparticles

MOLM13, RAW264.7, MV411 cells were seeded in 24-well plate (2 × 10^5^ per well) and incubated with Rhodamine B-labeled Membrane/Cu-HMPB NPs for 3 h. Then, the cells were washed with PBS and 100 µL cell suspensions (2 × 10^5^ cells mL^− 1^) were deposited onto the slides using Statspin Cytofuge 12 (1600 rpm, 4 min). The deposited cells were fixed with 4% paraformaldehyde and the cell nuclei were stained with 4′,6-diamidino-2-phenylindole. Then the nanoparticle uptake was observed under CLSM (Ex 595 nm, Em 570 nm). Cell uptake of the nanoparticles was also quantified using flow cytometry.

### Cell viability assay

The Cell Counting Kit-8 (CCK-8, Dojindo, Kumamoto, Japan) assay was performed to measure cell viability according to the manufacturer’s instructions. Briefly, the cells were cultured in 96-well plates, treated with drugs at the indicated concentrations, and then incubated with the CCK-8 working solution for 1–4 h at 37 °C. The resulting absorbance was detected at 450 nm in a microplate reader.

### In vitro and in vivo pH-responsiveT_1_-weighted MRI imaging

The longitudinal relaxation (T1) of homogeneous aqueous dispersions of Membrane/Cu-HMPB@DSF/RSL3 NPs with Mn^2+^ ion concentrations ranging from 3.9 to 62.5 µM was evaluated using a 3.5 T Bruker Biospec small animal MRI system (Bruker, Billerica, MA) at 25 ± 0.5 °C.

A leukemia mouse model was established by intravenously injecting 5 × 10⁵ C1498 cells into the tail vein of C57BL/6 mice (*n* = 1). 100 µL of Membrane/Cu-HMPB@DSF/RSL3 NPs suspensions in saline (10 mg mL⁻¹) was administered *via* tail vein injection to C57BL/6 mice. At 0, 0.5, 1, 2, 4, 6, 8, 10, 12, 24, and 48 h post-injection, mice were anesthetized with intraperitoneal 5% chloral hydrate (50 mg/kg) and imaged using a 3.0 T T1-weighted MRI system (Siemens MAGNETOM Prisma) with the following parameters: TR = 1820 ms, TE = 30 ms, FOV = 240 × 240 mm². The bone marrow region was selected as the region of interest (ROI) for quantitative T1 mapping. All animal experiments were conducted in accordance with institutional ethical guidelines.

### Anti-Leukemia therapy in the C1498 model mice

On day 7 after C1498 cell inoculation, mice were divided into five groups (*n* = 10) based on the bioluminescence intensity of whole body, and i.v. injected with the various formulations (PBS, DSF/RSL3, Cu-HMPB, Cu-HMPB@DSF/RSL3, and Membrane/Cu-HMPB@DSF/RSL3 NPs) once every three days. The mice bone marrow and spleens were excised, photographed, or weighted. C1498-GFP cells in BM were also analyzed by flow cytometry. Major organs (heart, liver, spleen, lung, and kidney) and femurs of the mice were processed H&E staining to identify the invasion of leukemic cells. The remaining mice in each group (*n* = 5–7) were monitored for survival assay. The increase in life span (ILS) was calculated from the survival data according to the following formula: % ILS = (T/C − 1) × 100%. T and C are the mean survival time of treated mice and control mice from the saline group, respectively.

### Anti-Leukemia therapy in the MLL-AF9 model mice

On day 7 after MLL-AF9 cell inoculation, mice were divided into five groups (*n* = 10) based on the bioluminescence intensity of whole body, and i.v. injected with the various formulations (PBS and Membrane/Cu-HMPB@DSF/RSL3 NPs) once every three days. The mice femurs and spleens were excised, photographed, or weighted. MLL-AF9 cells in BM were also analyzed by flow cytometry. Major organs (heart, liver, spleen, lung, and kidney) and femurs of the mice were processed H&E staining to identify the invasion of leukemic cells. The remaining mice in each group (*n* = 5–7) were monitored for survival assay. The increase in life span (ILS) was calculated from the survival data according to the following formula: % ILS = (T/C − 1) × 100% [42]. T and C are the mean survival time of treated mice and control mice from the saline group, respectively.

### Western blotting

The cells were harvested, washed with PBS, and lysed by 2⋅SDS buffer. Equal quantities of protein extract were electrophoresed using sodium dodecyl sulfate-polyacrylamide gel electrophoresis (SDS-PAGE) and transferred to nitrocellulose membranes. The membranes were blocked with 5% non-fat milk at room temperature for 1 h and incubated with primary antibodies overnight at 4 °C. The membranes were then incubated with the horseradish peroxidase (HRP)-conjugated secondary IgG antibody at room temperature for 2 h. The membranes were finally imaged using the ECL detection system (Thermo Fisher, USA). The antibodies used are as follows: CD135 (#3462T, Cell Signaling Technology), CD47 (#63000T. Cell Signaling Technology), GPX4 (30388-1-AP, Proteintech, China), FDX1 (12592-1-AP, Proteintech, China), LIAS (11577-1-AP, Proteintech, China), 4-HNE (NBP2-59353, Bio-Techne, USA) β-actin (#4970, Cell Signaling Technology), STING (66680-1-Ig., Proteintech, China), P-TBK (AP1418, ABclonal, China).

### Immunotherapy

In an ice box, the collected bone marrow were homogenized into a tissue suspension. The tissue suspension was filtered through a 70 μm cell strainer to obtain a single-cell suspension. To isolate lymphocytes, the suspension was treated with lymphocyte separation medium. Next, the samples were incubated with fluorescence-labeled antibodies according to the manufacturer’s protocol: APC anti-mouse CD86 (159215, BioLegend, Inc., USA), PE anti-mouse CD80 (104707, BioLegend, Inc., USA), PE anti-mouse CD3 (100205, BioLegend, Inc., USA), and APC/cyanine7 anti-mouse CD8b (126619, BioLegend, Inc., USA). The stained cells were analyzed using a flow cytometer. Quantification of ATP, LDH, immune cells and cytokine was performed using commercial ELISA kits, with all steps conducted according to the manufacturers’ instructions.

### Hemolysis assay

To assess the biosafety of Membrane/Cu-HMPB@DSF/RSL3 NPs, a hemolysis test was conducted using red blood cells (RBCs) extracted from fresh blood of C57BL/6 mice. The RBCs were immediately transferred to EDTA-anticoagulated tubes to prevent clotting and centrifuged at 3500 × g for 5 min at 4 °C. The pellets were rinsed three times with PBS (pH 7.4) until the supernatant was colorless. Subsequently, 100 µL of saline containing Membrane/Cu-HMPB@DSF/RSL3 NPs suspensions (50, 100, 200, 400, and 600 µg/mL) was added to 1 mL of RBC suspension and co-incubated at 4 °C for 30 min. The supernatant was collected by centrifugation at 3500 × g for 5 min, and the absorbance was measured at 540 nm using a UV-vis spectrophotometer. PBS and deionized water were used as the negative and positive controls, respectively. The experiment was repeated three times, and the hemolysis rate was calculated using the formula:

$$\mathrm{H}\mathrm{e}\mathrm{m}\mathrm{o}\mathrm{l}\mathrm{y}\mathrm{s}\mathrm{i}\mathrm{s}\text\,{r}\mathrm{a}\mathrm{t}\mathrm{e}\left(\mathrm{\%}\right)=\frac{{A}_{NPs}-{A}_{c}}{{A}_{p}-{A}_{c}}$$

where *A*_*NPs*_, *A*_*c*_, *A*_*p*_ are the absorbance values of the experimental group, negative control group and positive control group, respectively. All procedures were performed in triplicate, and data are presented as mean ± SD.

### Biosafety assessment

Before sacrificing the AML (C1498)-bearing mice, blood samples were collected via cardiac puncture into EDTA-anticoagulated tubes to prevent coagulation. The blood was immediately centrifuged at 3500 × g for 5 min at 4 °C, and the supernatant (serum) was carefully collected. Biochemical parameters, including aspartate aminotransferase (AST), alanine aminotransferase (ALT), blood urea nitrogen (BUN), and creatinine (CRE), were quantified using a commercial assay kit (Cat. No. C010-2-1, Nanjing jiancheng Bioengineering Institute, China) following the manufacturer’s instructions. All procedures were performed in triplicate, and data are presented as mean ± SD.

### Synergy calculations

Synergy data were analyzed with online software (https://synergyfinder.aittokallio.group/20260315100806483805) ZIP synergy scores over 10 were considered to be synergistic, ZIP synergy scores between 0 and 10 were considered to be additive, and ZIP synergy scores below 0 were considered antagonistic.

### Statistical analysis

All experiments were repeated 3–4 times, and data are presented as mean ± SD. Student’s t-test was used for pairwise comparisons between groups. Statistical analyses were performed using GraphPad Prism 5.0 (GraphPad Software, San Diego, CA). *P < 0.05, **P < 0.01, ***P < 0.001, ****P < 0.0001, * versus control group.

## Supplementary Information

Supplementary Material 1.

Supplementary Material 2.

## Data Availability

The materials generated in this study are available from the corresponding author upon request.
