# Supplementary material for: A degradable multi-metal-chelating stealth nanoplatform for dual ferroptosis/cuproptosis-enhanced metalloimmunotherapy in leukemia
Source: J Nanobiotechnology. 2026 Mar 21;24:399. doi: 10.1186/s12951-026-04295-4 (PMC13126918; doi:10.1186/s12951-026-04295-4)
Supplement: Supplementary file 1 — Supplementary Material 1. [file 12951_2026_4295_MOESM1_ESM.docx]

**Supplementary Information**

**Additional data Figures S1 to S29**

**
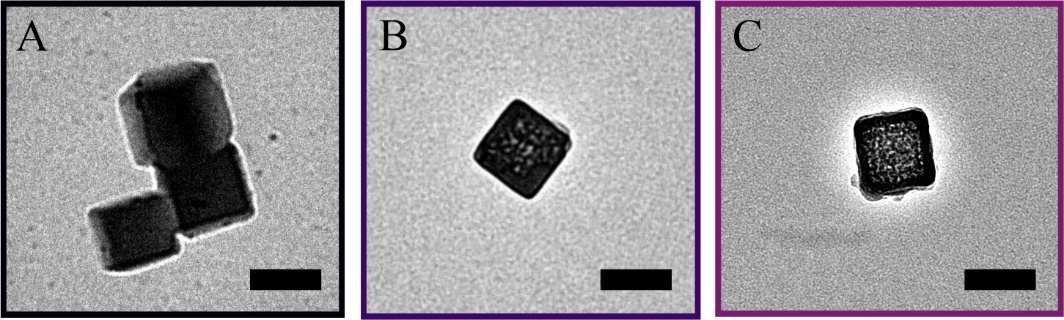
**

Figure S1. TEM images of (A) MPB NPs, (B) HMPB NPs and (C) Cu-HMPB NPs. (Scale bar: 100 nm).


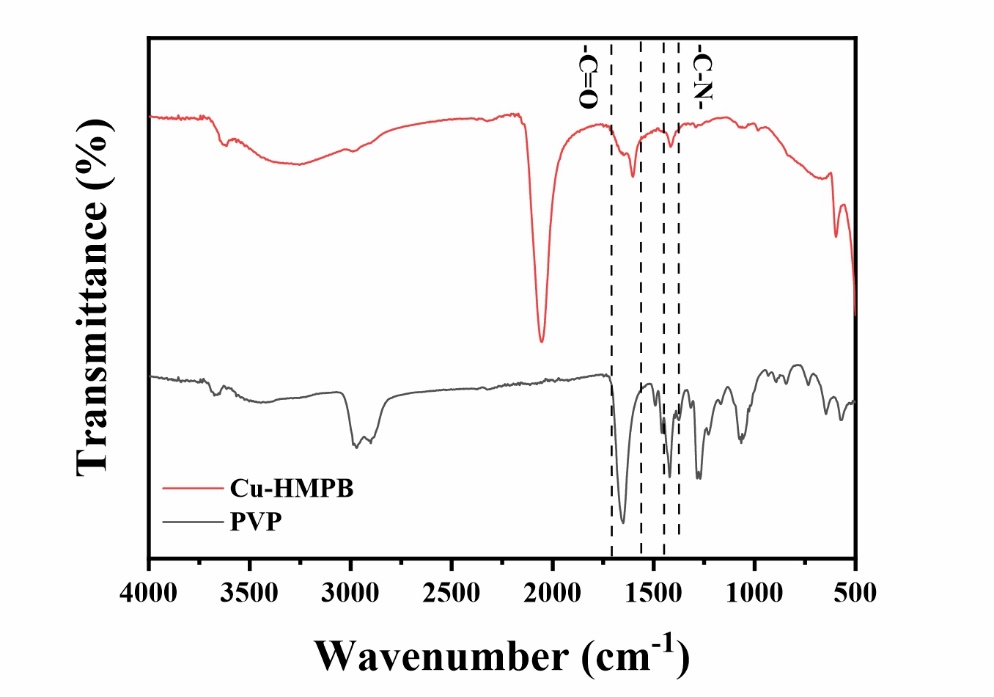


Figure S2. FT-IR spectra for PVP and Cu-HMPB NPs.


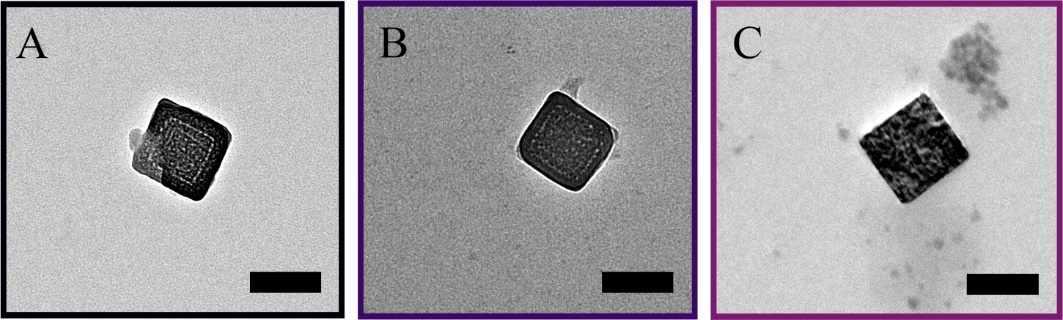


Figure S3. TEM images of (A) Cu-HMPB@DSF NPs, (B) Cu-HMPB@DSF/PAH NPs and (C) Cu-HMPB@DSF/RSL3 NPs. (Scale bar: 100 nm).


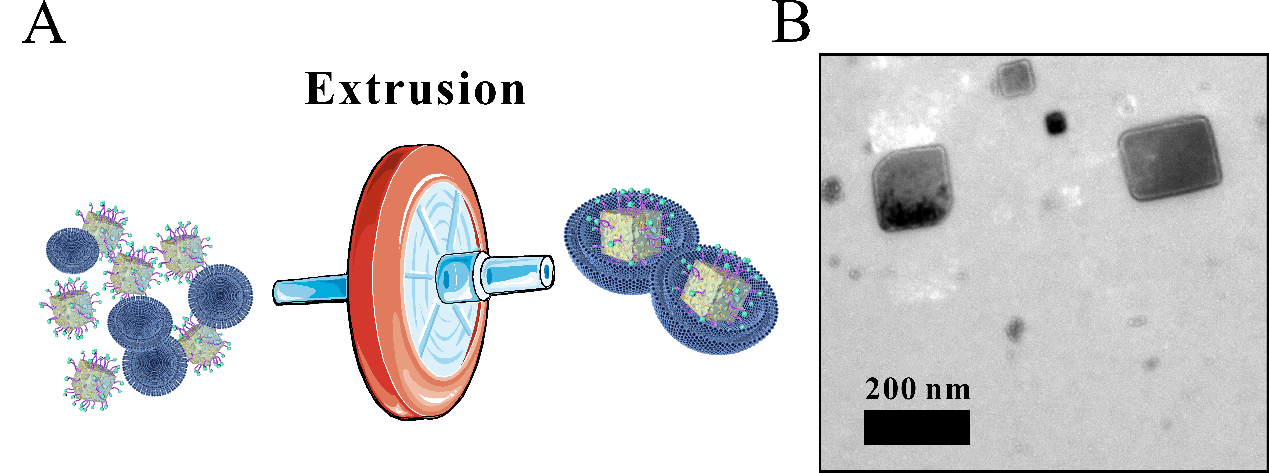


Figure S4. (A) Synthesis of Membrane/Cu-HMPB@DSF/RSL3 NPs. (B) TEM image of Membrane/Cu-HMPB@DSF/RSL3 NPs. Scale bar: 200 nm.


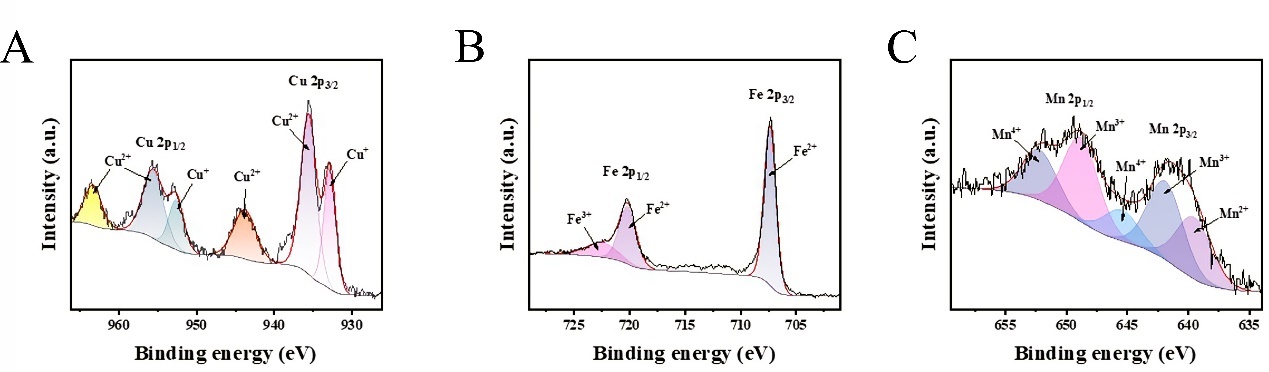


Figure S5. (A) Cu 2p, (B) Fe 2p and (C) Mn 2p XPS high-resolution spectrum of Membrane/Cu-HMPB@DSF/RSL3 NPs.


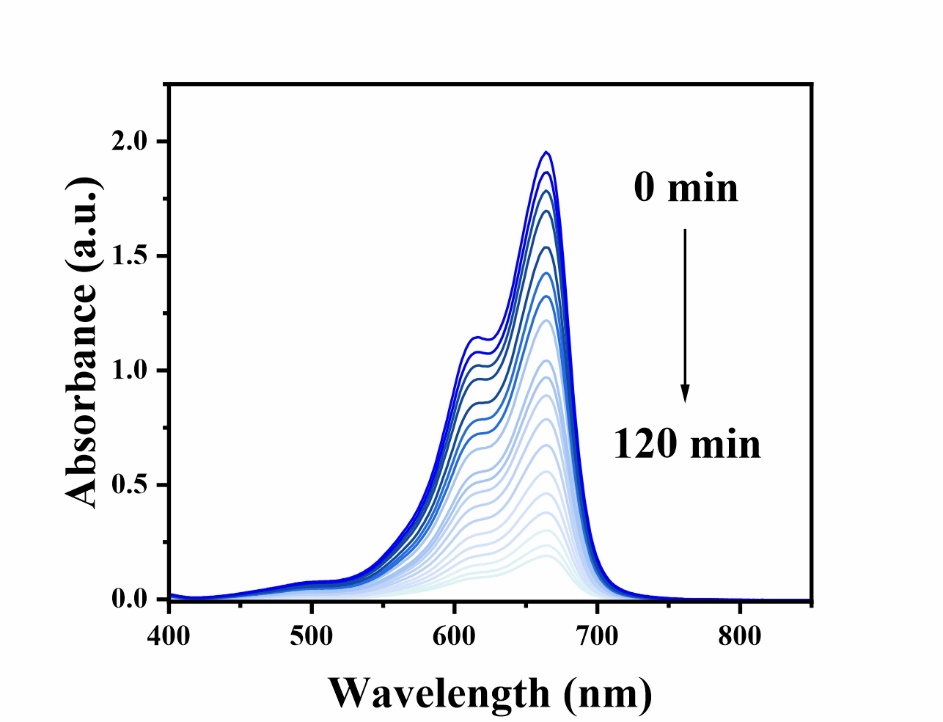


Figure S6. UV-visible adsorption spectra showing MB degradation by the Membrane/Cu-HMPB@DSF/RSL3 NPs for different reaction time.


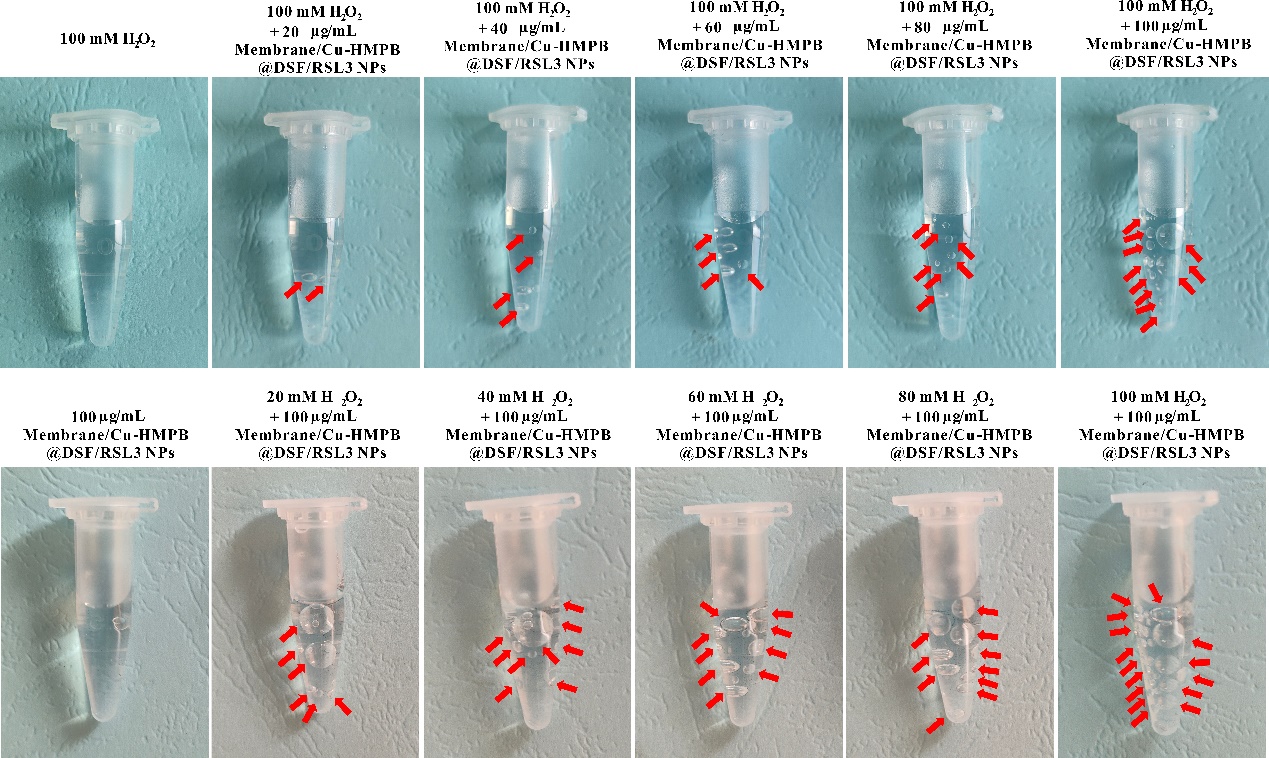


Figure S7. Photographs of bubbles present in catalase-like reactions catalyzed by the Membrane/Cu-HMPB@DSF/RSL3 NPs after 24 h incubation. Red arrows indicate bubbles.


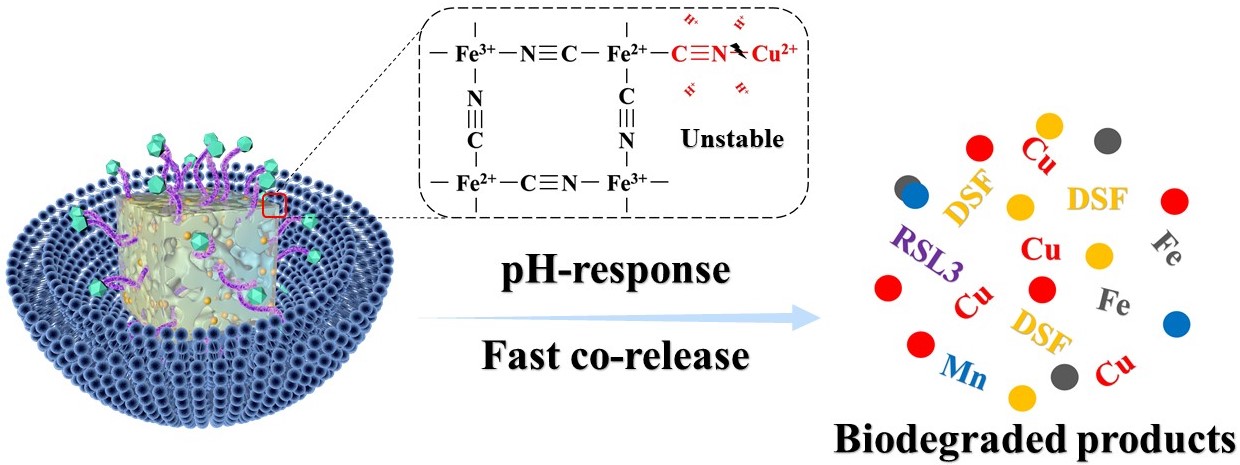


Figure S8. Schematic illustration of the pH-responsive degradation and co-release behavior of Membrane/Cu-HMPB@DSF/RSL3 NPs.


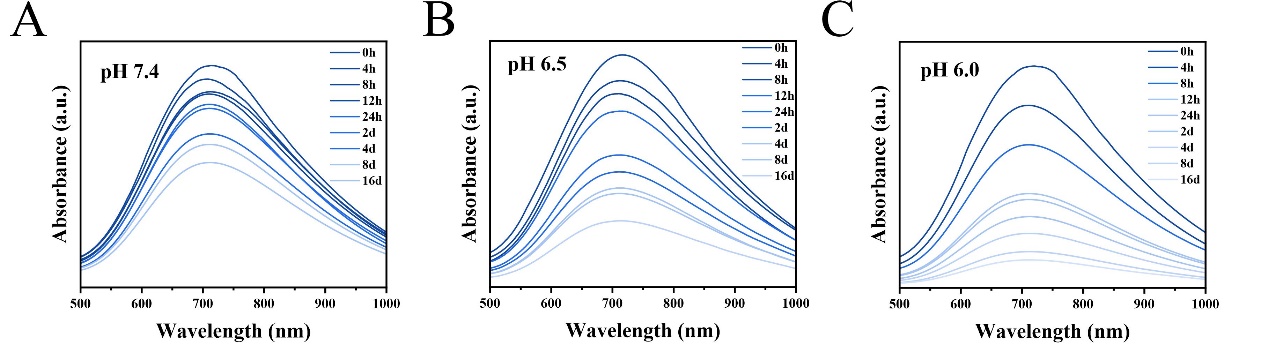


Figure S9. (A-C) Vis-visible absorbance spectra of Membrane/Cu-HMPB@DSF/RSL3 NPs at the range of 500-1000 nm after immersing in (A) (pH 7.4), (B) (pH 6.5) and (C) (pH 6.0) SBF for different reaction times.


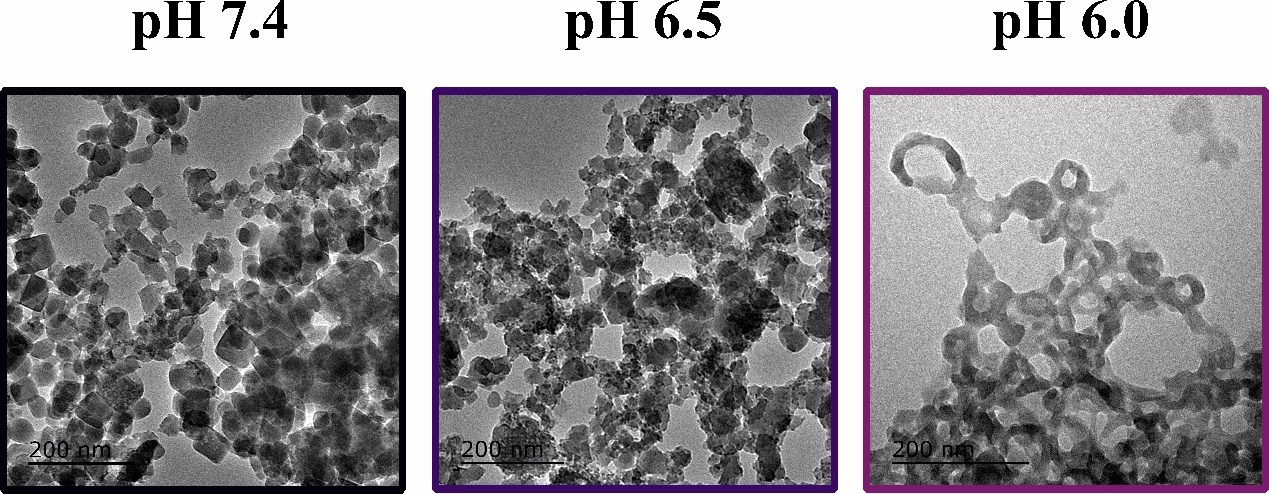


Figure S10. TEM images of Membrane/Cu-HMPB@DSF/RSL3 NPs immersing SBF solution at various pH values for 4 days.


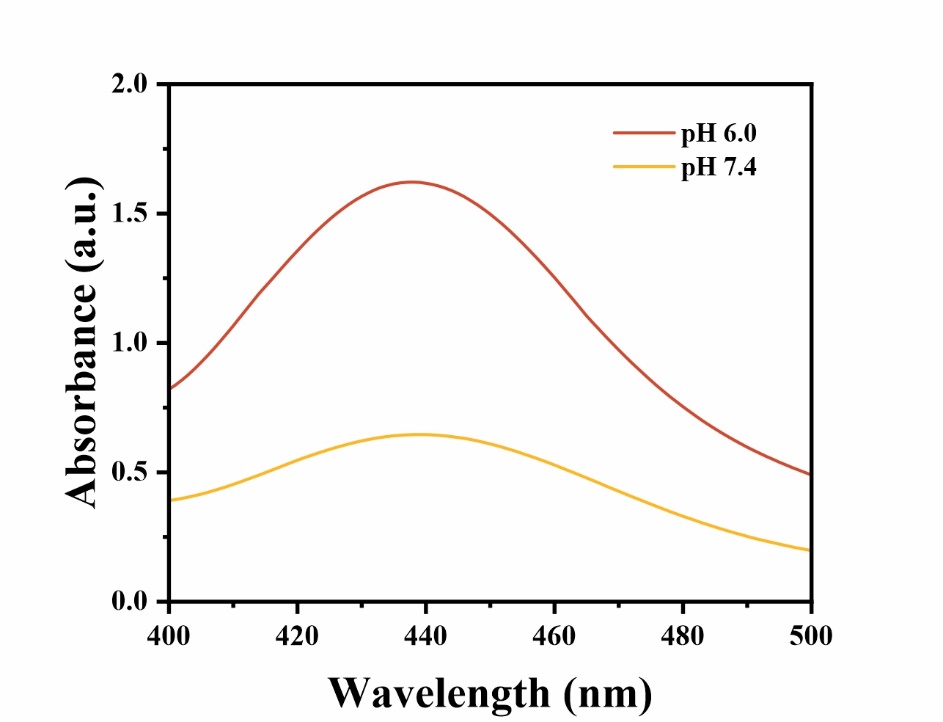


Figure S11. UV-Visible absorbance spectra of Membrane/Cu-HMPB@DSF/RSL3 NPs at the range of 400-500 nm in SBF (pH 7.4 or 6.0) solution for 1 day.


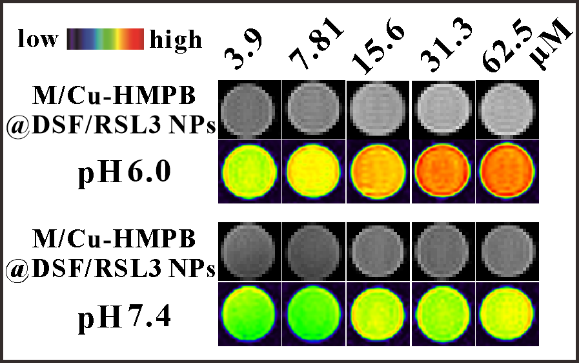


Figure S12. *T*_1_-weighted MRI images of Membrane/Cu-HMPB@DSF/RSL3 NPs in pH 7.4 and 6.0 aqueous solutions at different concentrations of iron ions: 3.9 μM, 7.81 μM, 15.6 μM, 31.3 μM, 62.5 μM.


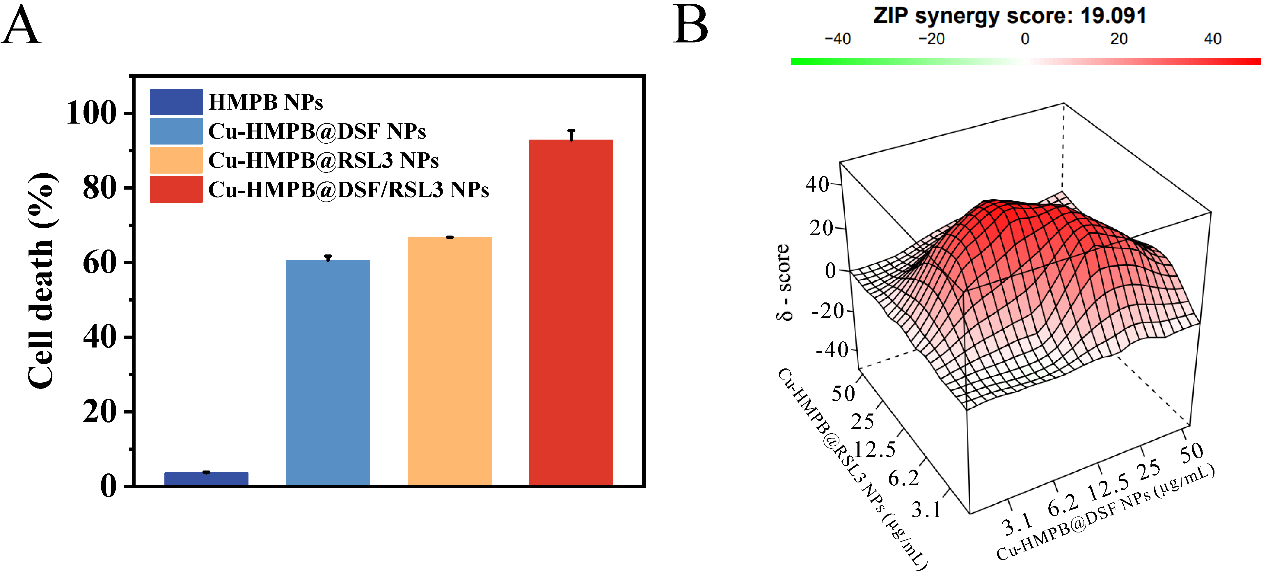


Figure S13. (A) Cell death after treatment with 50 μg/mL HMPB NPs, Cu-HMPB@DSF NPs, Cu-HMPB@RSL3 NPs or Cu-HMPB@DSF/RSL3 NPs. The data are shown as mean ± SD (n = 3). (B) The cell viability was determined by the CCK-8 kit. Data were analyzed online (https://synergyfinder.fimm.fi).


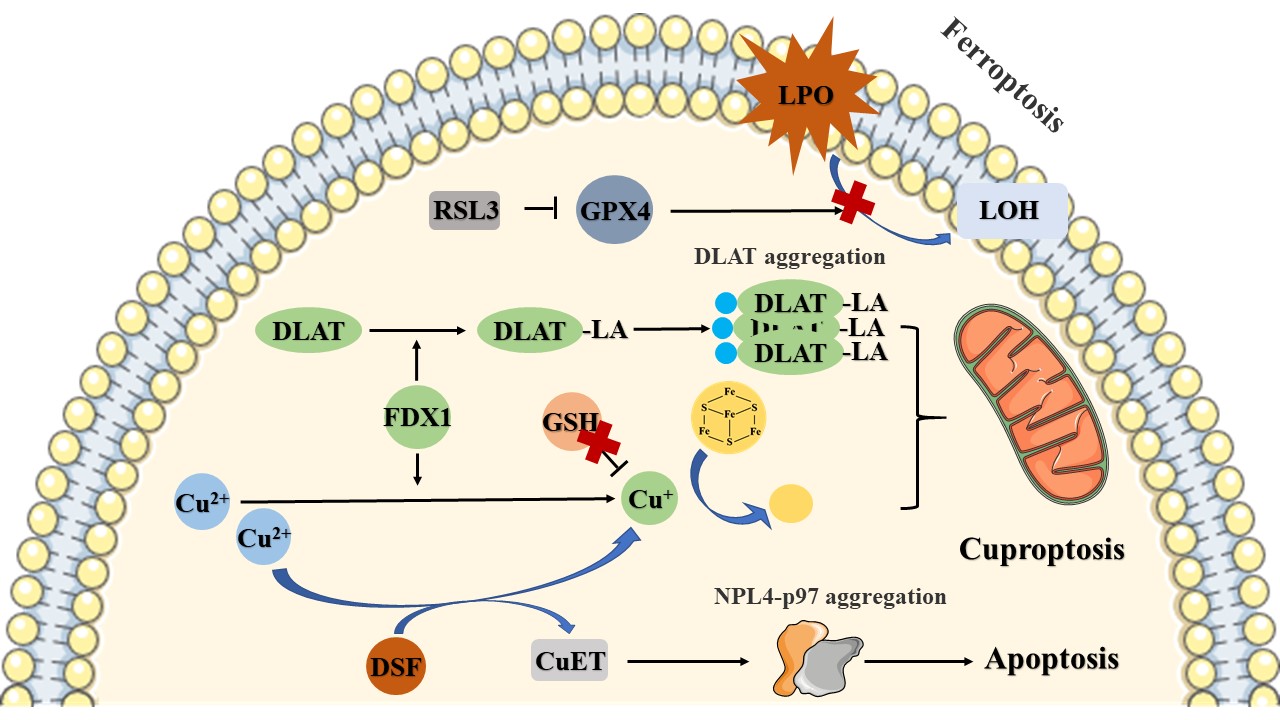


Figure S14. Schematic of the mechanism of apoptosis, ferroptosis and cuproptosis caused by Membrane/Cu-HMPB@DSF/RSL3 NPs.


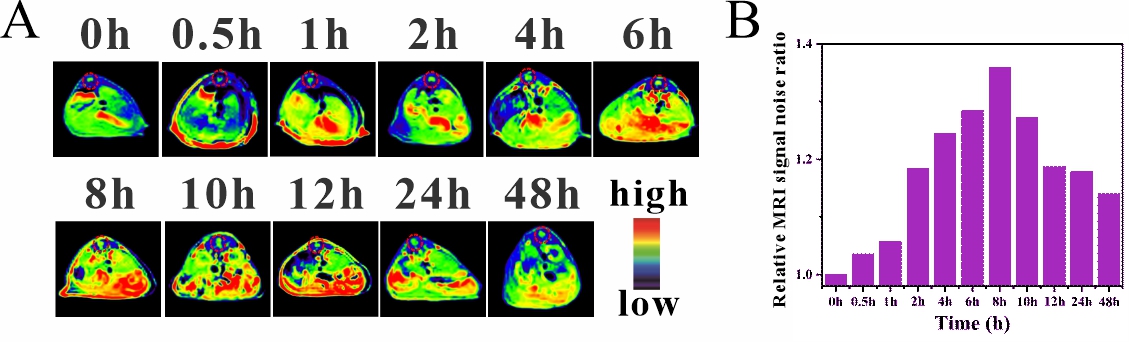


Figure S15. (A) MRI images. (the red circles denote the bone marrow site). (B) Relative MRI signal noise ratio.


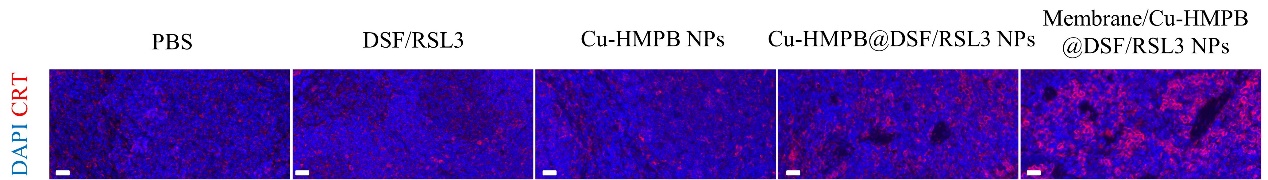


Figure S16. CRT staining of the spleen tissue. Scale bars = 20 μm.


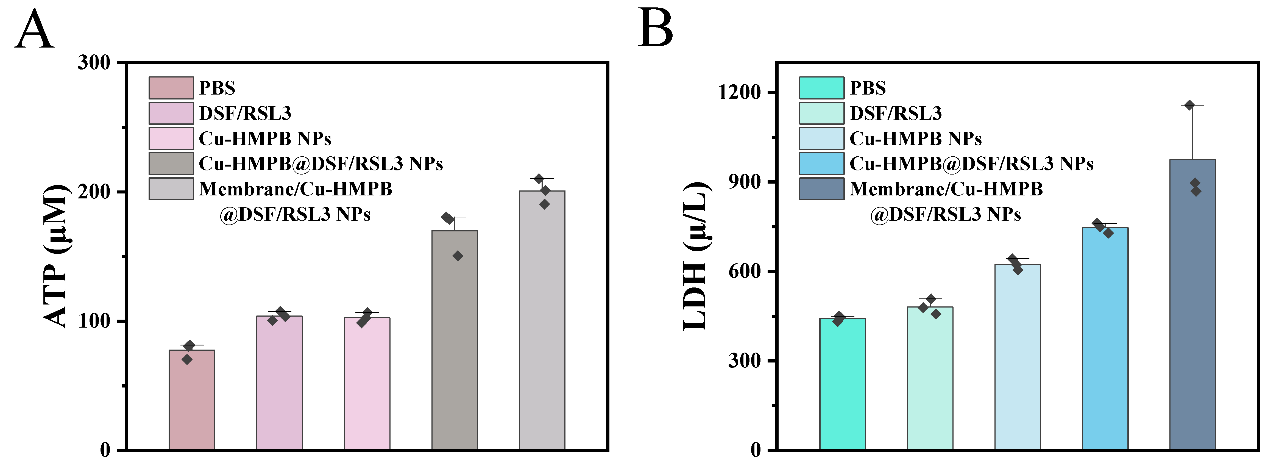


Figure S17. ELISA quantification of ATP (A) and LDH (B) levels in the bone marrow.


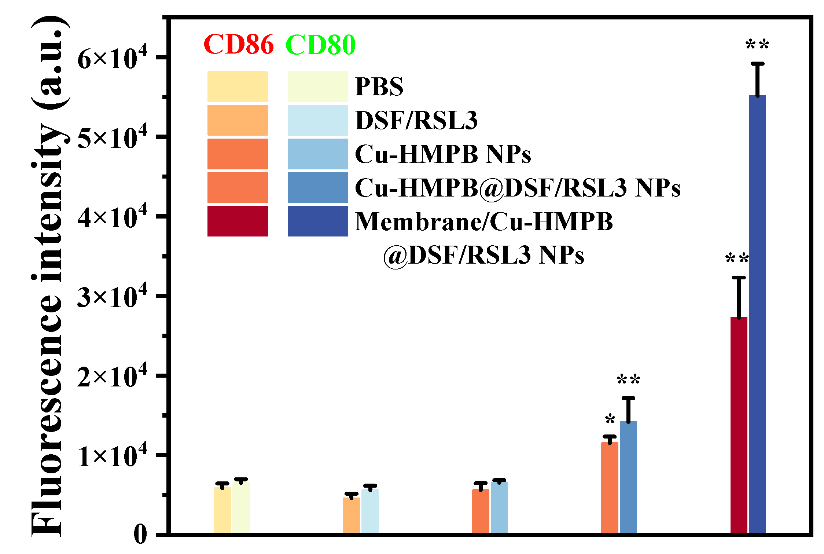


Figure S18. Quantitative analysis of the DCs intracellular fluorescence intensities in Figure 5H.


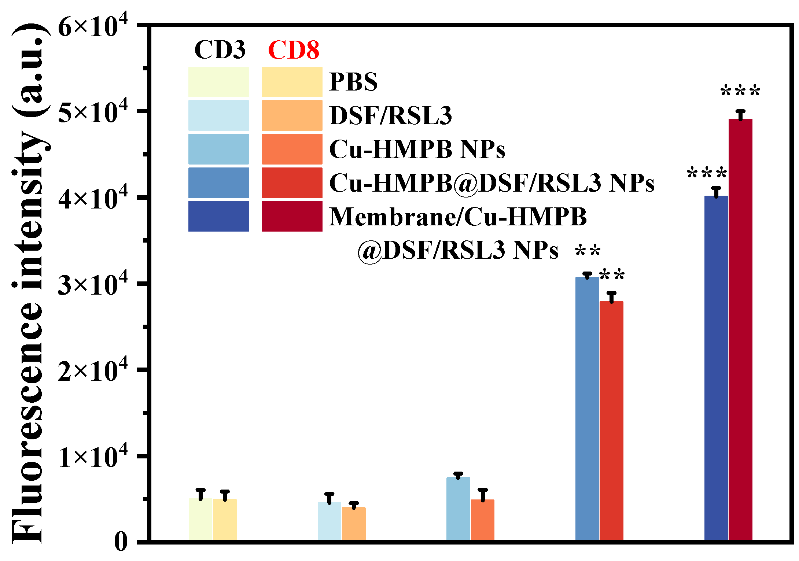


Figure S19. Quantitative analysis of the CTLs intracellular fluorescence intensities.


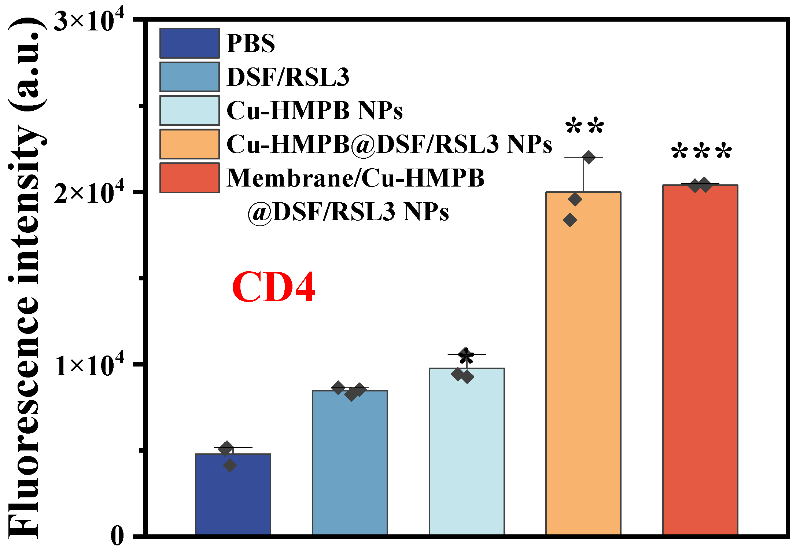


Figure S20. Quantitative analysis of the helper-T cells intracellular fluorescence intensities.


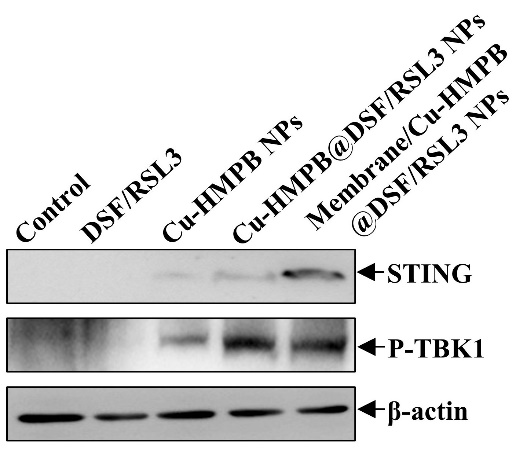


Figure S21. Expression levels of STING and p-TBK1 in the spleen tissue.


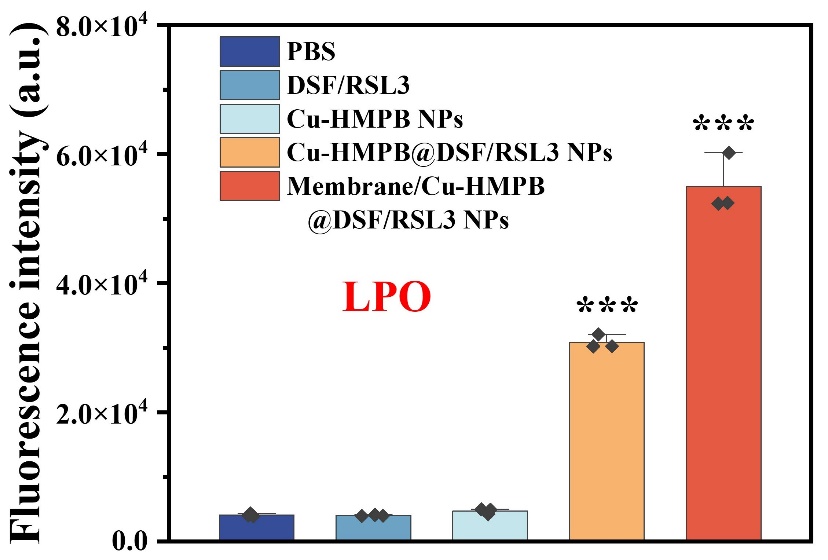


Figure S22. Quantitative analysis of the intracellular fluorescence intensities of LPO in spleen.


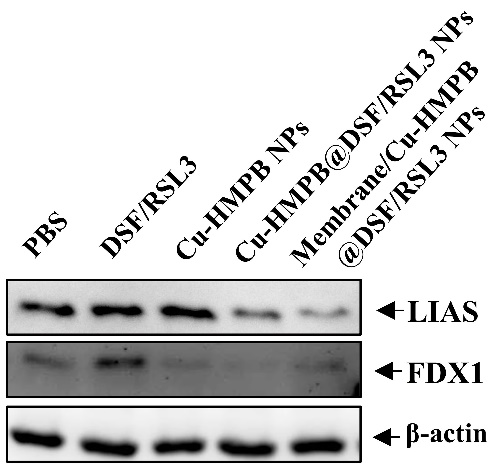


Figure S23. Expression levels of LIAS and FDX1 in the spleen tissue of mice from different treatment groups.


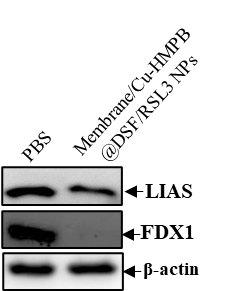


Figure S24. Expression levels of LIAS and FDX1 in the spleen tissue.


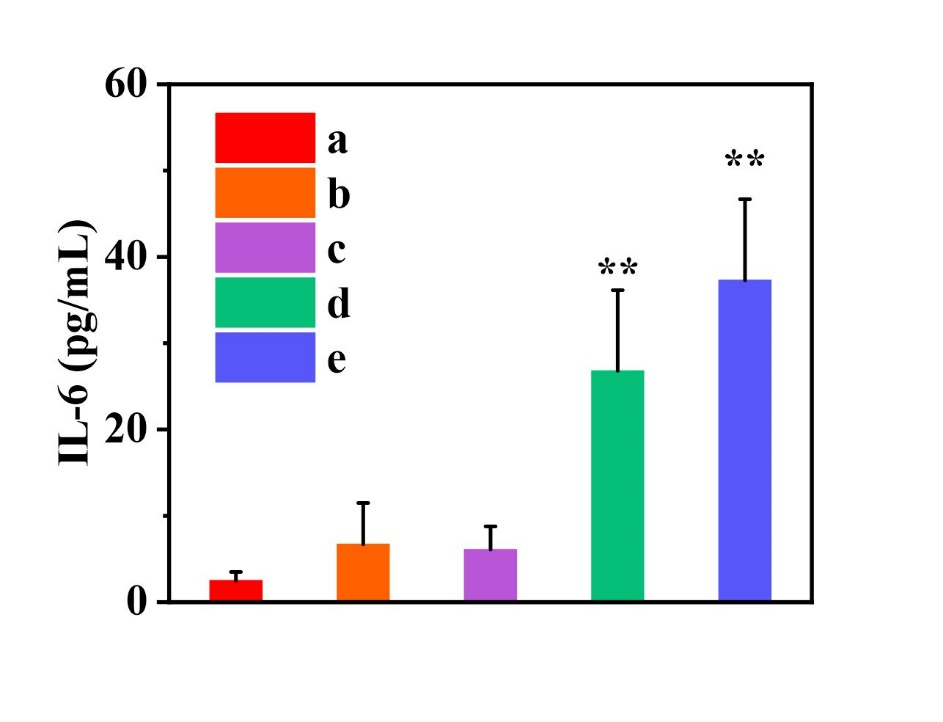


Figure S25. ELISA quantification of IL-6 levels in different treatment groups.


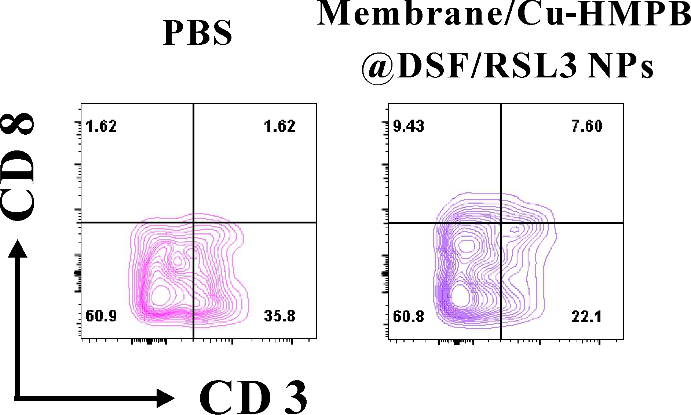


Figure S26. FCM plots of CTLs in bone marrow tissues from MLL-AF9 AML model mice.


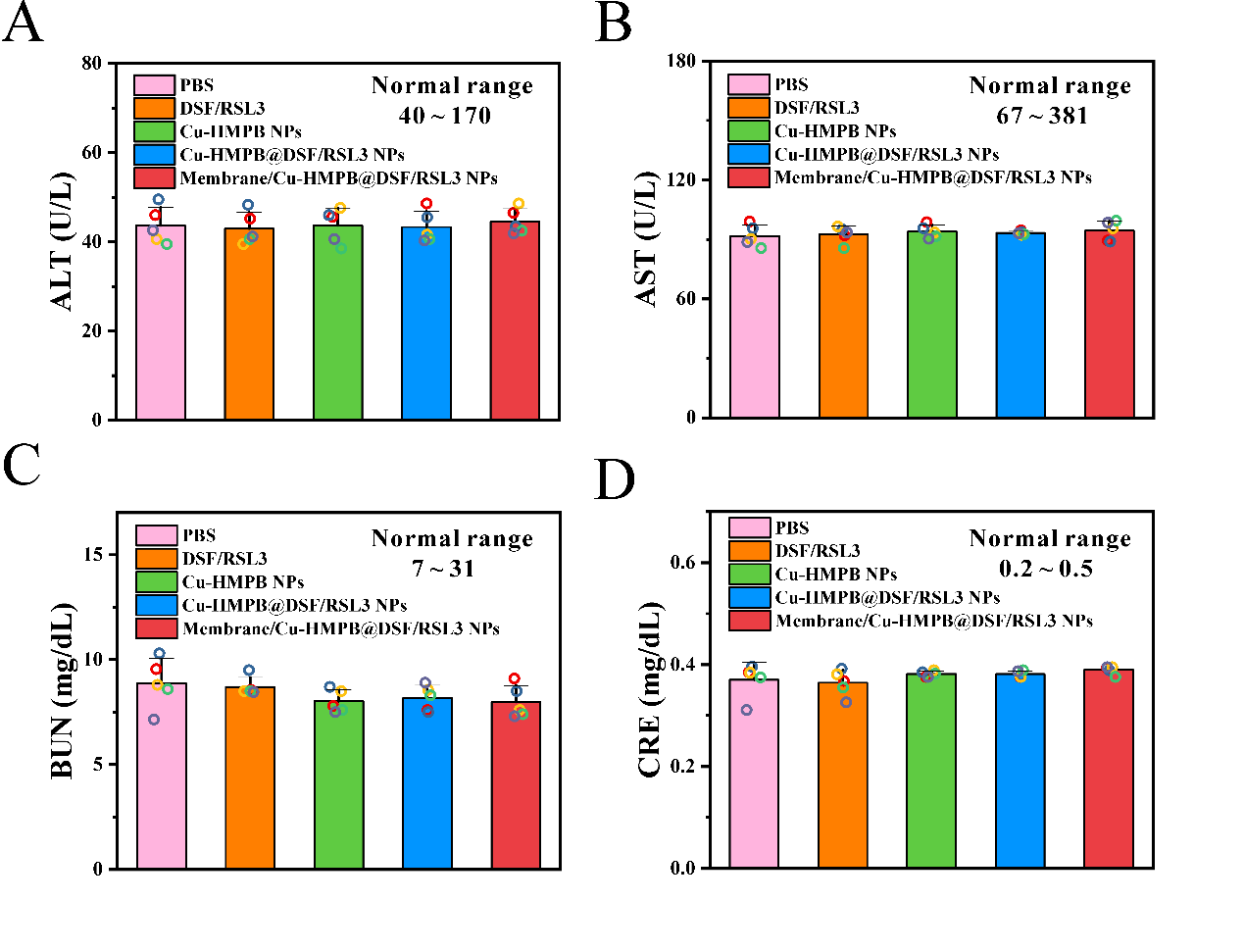


Figure S27. Blood biochemical analysis performed 30 days after the start of the treatment regimen in the C1498-bearing mouse model.


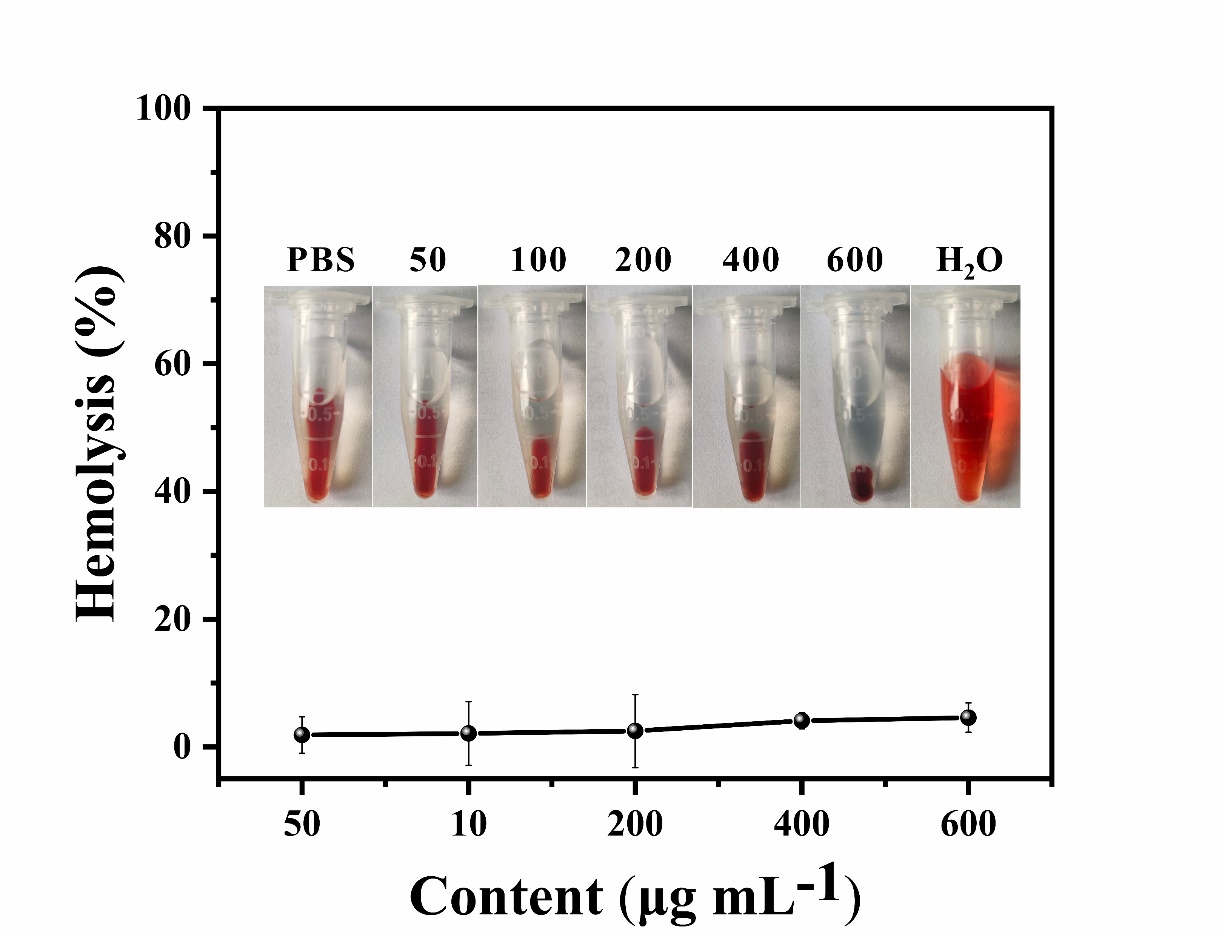


Figure S28. Hemolysis measurements performed with mouse red blood cells upon treatment with Membrane/Cu-HMPB@DSF/RSL3 NPs.


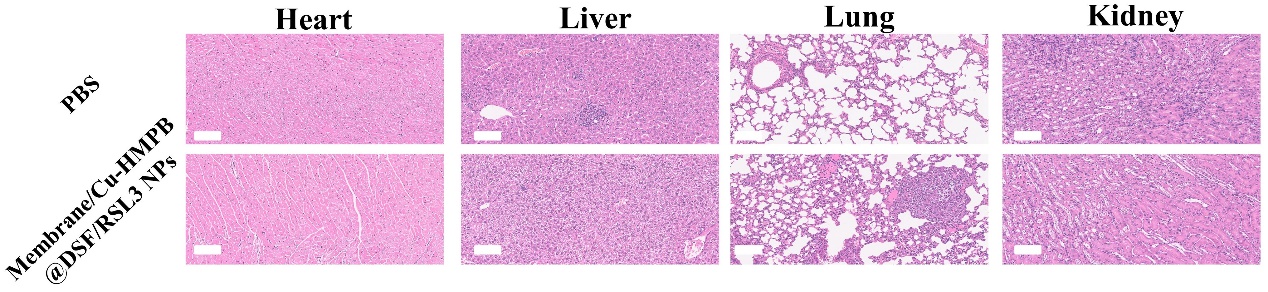


Figure S29. H&E staining images of heart, spleen, lung and kidneys from control (saline) or Membrane/Cu-HMPB@DSF/RSL3 NPs treated mice (scale bars=200 μm).

Table S1 The content of each element of Membrane/Cu-HMPB@DSF/RSL3 NPs, as determined by ICP-OES. The data are presented as the mean ± SD.

| Content (μg/mg) | Fe | Cu | Mn | S |
| --- | --- | --- | --- | --- |
|  | 76.29±1.9 | 27±0.76 | 10.6±0.27 | 42.99±0.62 |
