# Supplementary figures and images for: A degradable multi-metal-chelating stealth nanoplatform for dual ferroptosis/cuproptosis-enhanced metalloimmunotherapy in leukemia
Source: J Nanobiotechnology. 2026 Mar 21;24:399. doi: 10.1186/s12951-026-04295-4 (PMC13126918; doi:10.1186/s12951-026-04295-4)

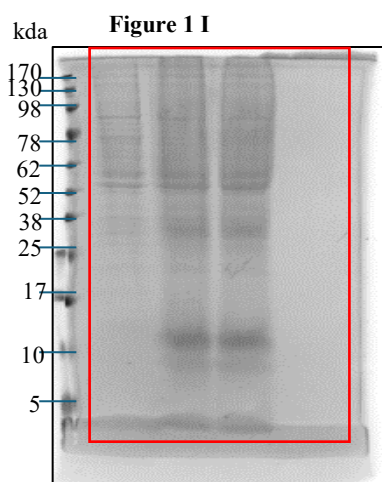

**Figure 1 J**

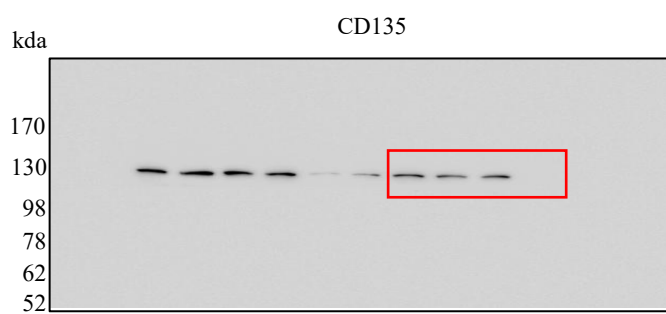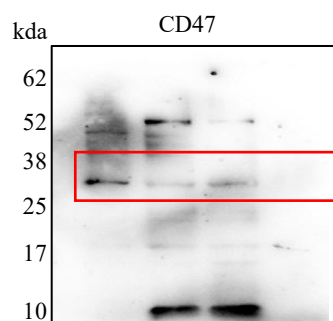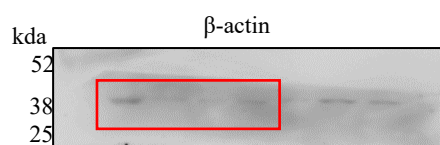

Figure 4 L

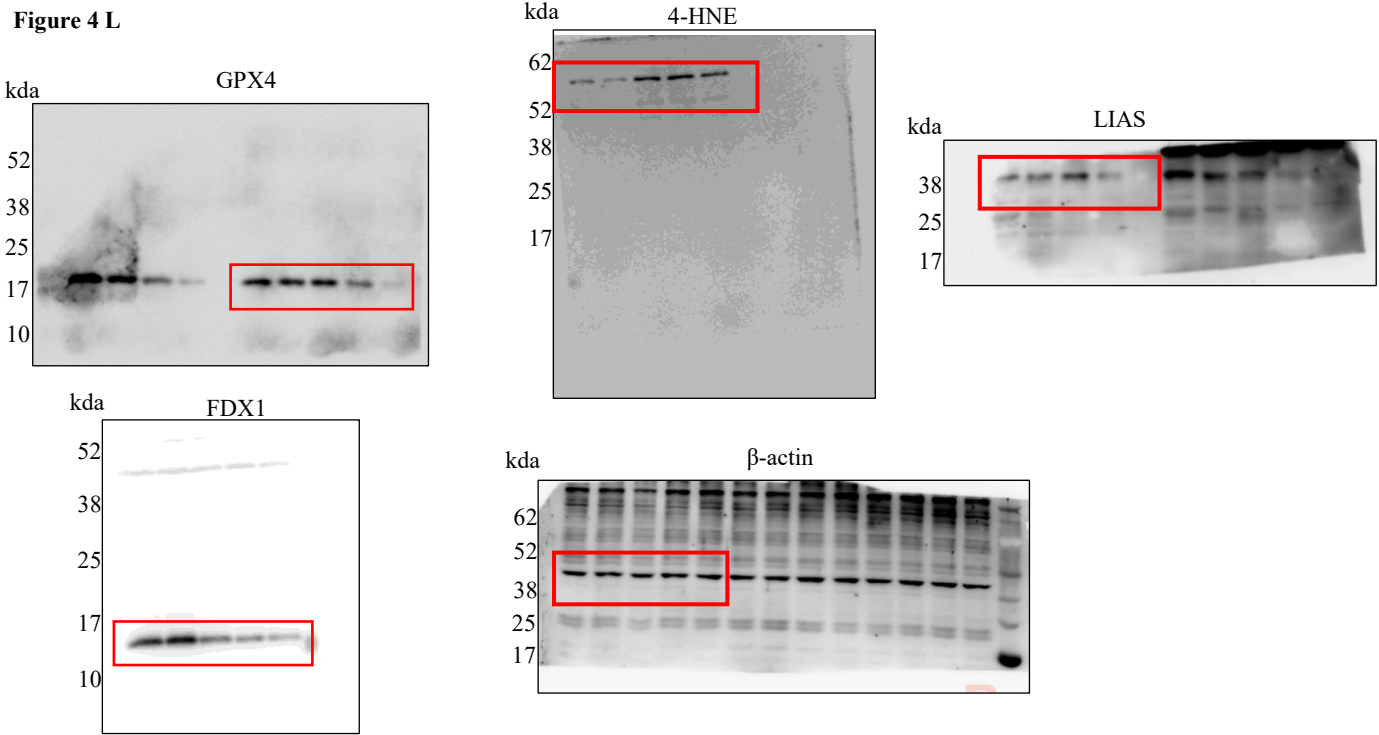

Figure 4 M

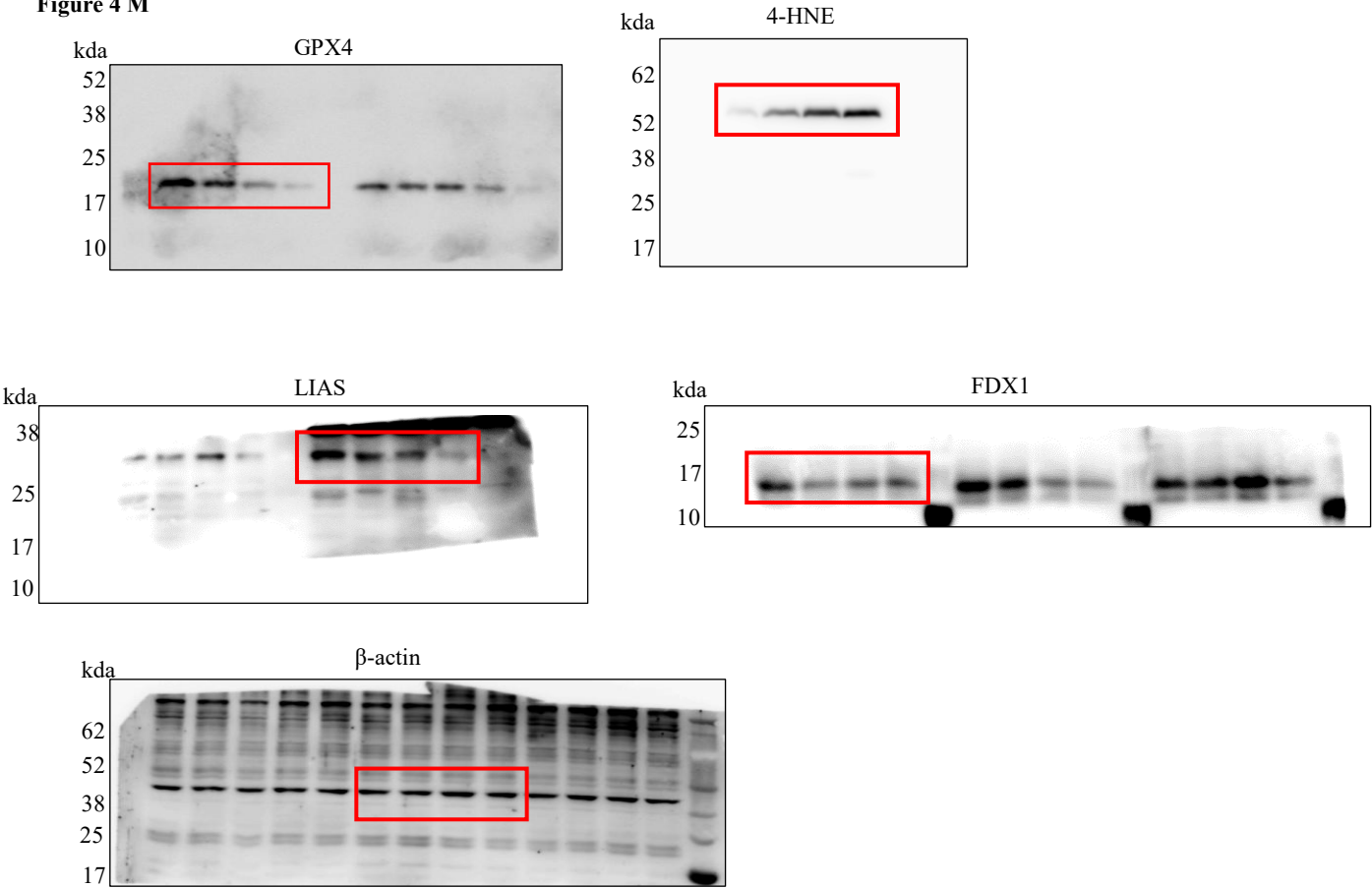

Figure 6G

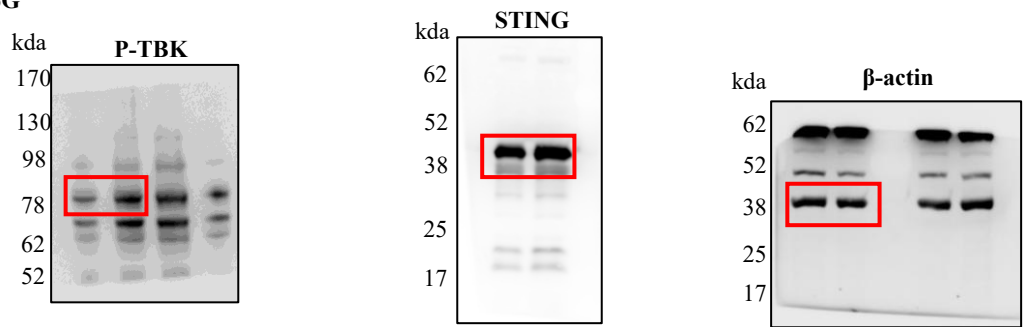

Figure S21

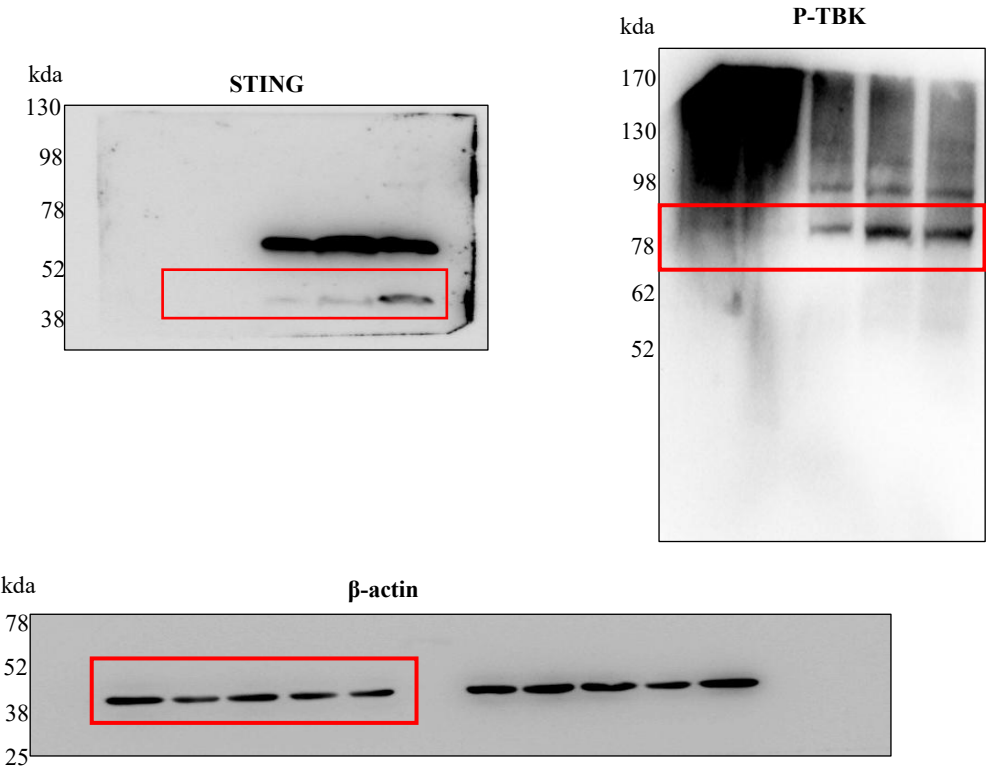

Figure S23

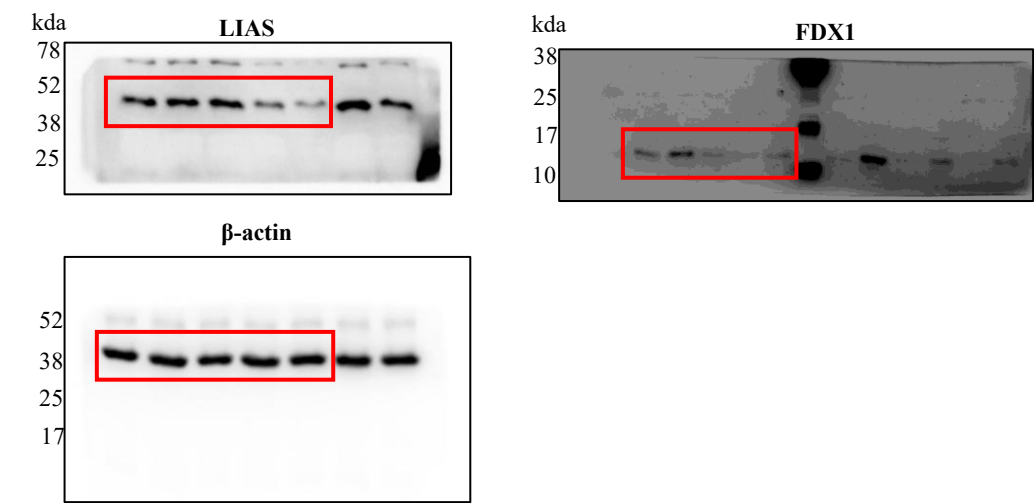

Figure S24

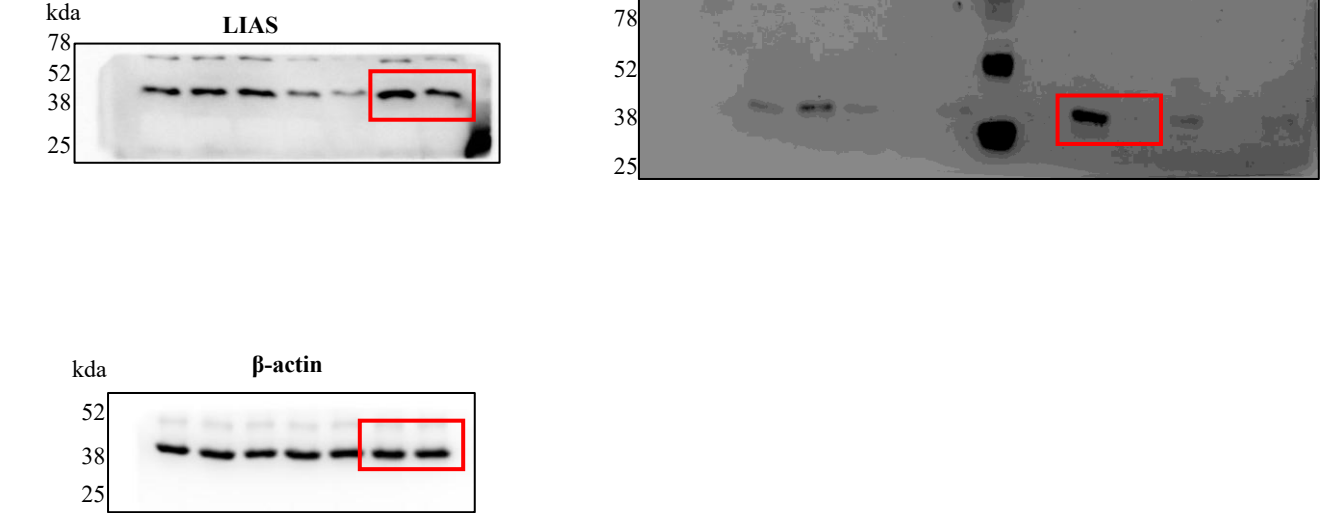

Supplement: Supplementary file 2 — Supplementary Material 2. [file 12951_2026_4295_MOESM2_ESM.pdf]
